# Dissecting mutational allosteric effects in alkaline phosphatases associated with different Hypophosphatasia phenotypes: An integrative computational investigation

**DOI:** 10.1371/journal.pcbi.1010009

**Published:** 2022-03-23

**Authors:** Fei Xiao, Ziyun Zhou, Xingyu Song, Mi Gan, Jie Long, Gennady Verkhivker, Guang Hu

**Affiliations:** 1 Center for Systems Biology, Department of Bioinformatics, School of Biology and Basic Medical Sciences, Soochow University, Suzhou, China; 2 Department of Chemistry, Multiscale Research Institute of Complex Systems and Institute of Biomedical Sciences, Fudan University, Shanghai, China; 3 Department of Computational and Data Sciences, Chapman University, One University Drive, Orange, California, United States of America; 4 Department of Biomedical and Pharmaceutical Sciences, Chapman University Pharmacy School 9401 Jeronimo Rd, Irvine, California, United States of America; Bogazici University, TURKEY

## Abstract

Hypophosphatasia (HPP) is a rare inherited disorder characterized by defective bone mineralization and is highly variable in its clinical phenotype. The disease occurs due to various loss-of-function mutations in *ALPL*, the gene encoding tissue-nonspecific alkaline phosphatase (TNSALP). In this work, a data-driven and biophysics-based approach is proposed for the large-scale analysis of *ALPL* mutations-from nonpathogenic to severe HPPs. By using a pipeline of synergistic approaches including sequence-structure analysis, network modeling, elastic network models and atomistic simulations, we characterized allosteric signatures and effects of the *ALPL* mutations on protein dynamics and function. Statistical analysis of molecular features computed for the *ALPL* mutations showed a significant difference between the control, mild and severe HPP phenotypes. Molecular dynamics simulations coupled with protein structure network analysis were employed to analyze the effect of single-residue variation on conformational dynamics of TNSALP dimers, and the developed machine learning model suggested that the topological network parameters could serve as a robust indicator of severe mutations. The results indicated that the severity of disease-associated mutations is often linked with mutation-induced modulation of allosteric communications in the protein. This study suggested that *ALPL* mutations associated with mild and more severe HPPs can exert markedly distinct effects on the protein stability and long-range network communications. By linking the disease phenotypes with dynamic and allosteric molecular signatures, the proposed integrative computational approach enabled to characterize and quantify the allosteric effects of *ALPL* mutations and role of allostery in the pathogenesis of HPPs.

## Author summary

By focusing on Hypophosphatasia, a rare inherited disorder we performed a comprehensive computational analysis of mutational effects on protein function in the encoded protein of Tissue Nonspecific Alkaline Phosphatase. This analysis demonstrated that pathogenic mutations can often modulate long-range allosteric interactions and communications in the dynamic protein network and the mechanisms underlying severity of mutational effects can be rationalized based on molecular principles of allostery. We have also developed a machine learning-based method to classify different disease phenotypes, and the interpretability of the classification model was addressed using the structural-functional analysis of network topologically important mutations. The results of this study revealed allosteric molecular signatures of severe mutations, suggesting that allosteric models of protein dynamics and function can be useful in dissecting complex genotype-phenotype relationships.

## Introduction

Hypophosphatasia (HPP) is a rare autosomal dominant or recessive metabolic disorder that constitutes a complex, multisystemic disease [1,2]. The clinical manifestation of this disease is highly diverse and is often linked with the mutational landscape and the inheritance mechanism [2]. From a prenatal lethal form with no skeletal mineralization to a mild form with late adult onset, HPP can be classified into six subtypes including perinatal, infantile, prenatal benign, childhood, adult and odonto [3]. In general, perinatal and infantile subtypes represent severe forms of HPP, while childhood, adult, odonto, and prenatal benign subtypes constitute mild phenotypes.[4] HPP is caused by loss-of-function mutations in the *ALPL* gene encoding the Tissue Nonspecific Alkaline Phosphatase (TNSALP).[5] TNSALP is a membrane-bound metalloenzyme, whose activity is reduced by various mutations in the *ALPL* gene, leading to the increased inorganic pyrophosphate, which in turn causes different HPP phenotypes [6]. As an enzyme replacement therapy, ENB-0040 is a bone-targeted, recombinant human TNSALP that prevents the manifestations of HPP [7]. However, to date, there is no established treatment for HPPs [8], due to the little knowledge about the relationship between mutations in the gene responsible for HPPs and phenotypic variability.

The *ALPL* gene is localized on chromosome 1p36.1–34 and consists of 12 exons distributed over 50 kb [9,10], *ALPL* mutation detection is important for recurrence risk assessment and prenatal diagnosis [11]. To date, more than 400 *ALPL* gene mutations have been reported worldwide and approximately 80% of these mutations are missense. Although HPP is caused by homozygous, heterozygous, or compound heterozygous *ALPL* mutations [12], most of these mutations cause changes in a single amino acid in TNSALP. Mutations in *ALPL* would reduce the enzyme activity of TNSALP mutant proteins to a varying degree, with residual activities often exhibiting different enzymatic properties from wild-type TNSALP. By measuring the effects of amino acid changes on TNSALP dimer stability, the relationship between *ALPL* missense variants and TNSALP enzymatic activity can be predicted [13]. Despite recent progress, the correlation between these mutations and the six HPP subtypes is not well established, and thus the molecular mechanisms underlying the genotypic-phenotypic relationships of HPP remain unclear [14]. A more detailed molecular characterization of *ALPL* mutations can help to better understand mechanisms driving the pathogenesis, and onset of HPP. In addition, understanding and characterization of the molecular determinants of pathogenic mutations may enhance a toolkit for inhibiting TNSALP and design of targeted therapies [15].

TNSALP is a homodimeric protein that contains several known domains, including five principal functional domains: catalytic site, calcium-binding site, crown domain, homodimer interface, and N-terminal alpha helix [12]. The assignment of various mutations to the five functional domains of the TNSALP structure model has contributed to the current understanding of the genotypic and phenotypic interrelationship of HPP [16]. For example, most of the severe missense mutations were localized in crucial domains, such as the active site, the vicinity of the active site, and the homodimer interface. The structural importance of the crown domain has also been highlighted, with respect to the catalytic function of TNSALP [17]. In addition, there is structural evidence to support the concept that these crucial domains are also involved in the allosteric properties of TNSALP, since the catalytic activity depends on its homodimeric configuration from which the dominant negative effect of some loss-of-function ALPL mutations is derived. Although mammalian alkaline phosphatases have been known as allosteric enzymes for many years [18], the dynamics-driven allosteric signaling pathways have yet to be investigated at an atomistic level. Therefore, the elucidation of the conformational dynamics and allosteric signatures of TNSALP caused by *ALPL* mutations would provide greater insight into the genotype-phenotype relationship in HPP [19,20].

The catalytic efficiency[21] and potential pathogenicity of mutations [22,23] are deeply interlinked with the molecular mechanisms and can be examined using evolutionary, structural and dynamics perspectives [24]. The disease-causing mutations frequently involve a drastic change in amino acid physicochemical properties, such as charge, hydrophobicity, and geometry, and are less surface exposed than polymorphic mutations [25]. In this study, we employ an integrative computational strategy to explore and quantify the molecular effects of *ALPL* mutations that combine structural analysis, biophysical simulations and modeling of dynamic interaction networks. Structural modeling combined with network theory has been widely exploited in studying protein topology, dynamics and allostery [26–28]. Dynamic network modeling of conformational ensembles has been proven as an efficient approach to describe structural and dynamic changes associated with mutational effects in complex protein systems [29–32]. Central to these approaches is characterization of conformational protein dynamics and mutation-induced changes in the protein equilibrium ensembles which is fundamental to understanding the physical basis of the effects of missense variants [33,34]. Elastic network models (ENMs) are widely recognized as robust simplified models of protein topology and dynamics [35,36] that have been successfully used to predict the effects of single-point mutations on protein stability [37]. By incorporating dynamic descriptors based on ENM with sequence- and structure-dependent properties, the prediction accuracy of the impact of variants on biological function has greatly increased [38]. Machine learning models that integrate sequence, structure, and ensemble-based features have been developed to classify mutation types [39–41]. Furthermore, biophysical simulations combined with structure-based modeling of residue interaction networks have also been used to reveal the functional role of mutation hotspots in molecular communication in some tumor suppressor proteins [42], regulatory complexes including HSp90 [43], and SARS-CoV-2 spike Protein [44], and classify *PTEN* missense variants corresponding to cancer or autism spectrum disorder [45–47].

**Fig 1:**
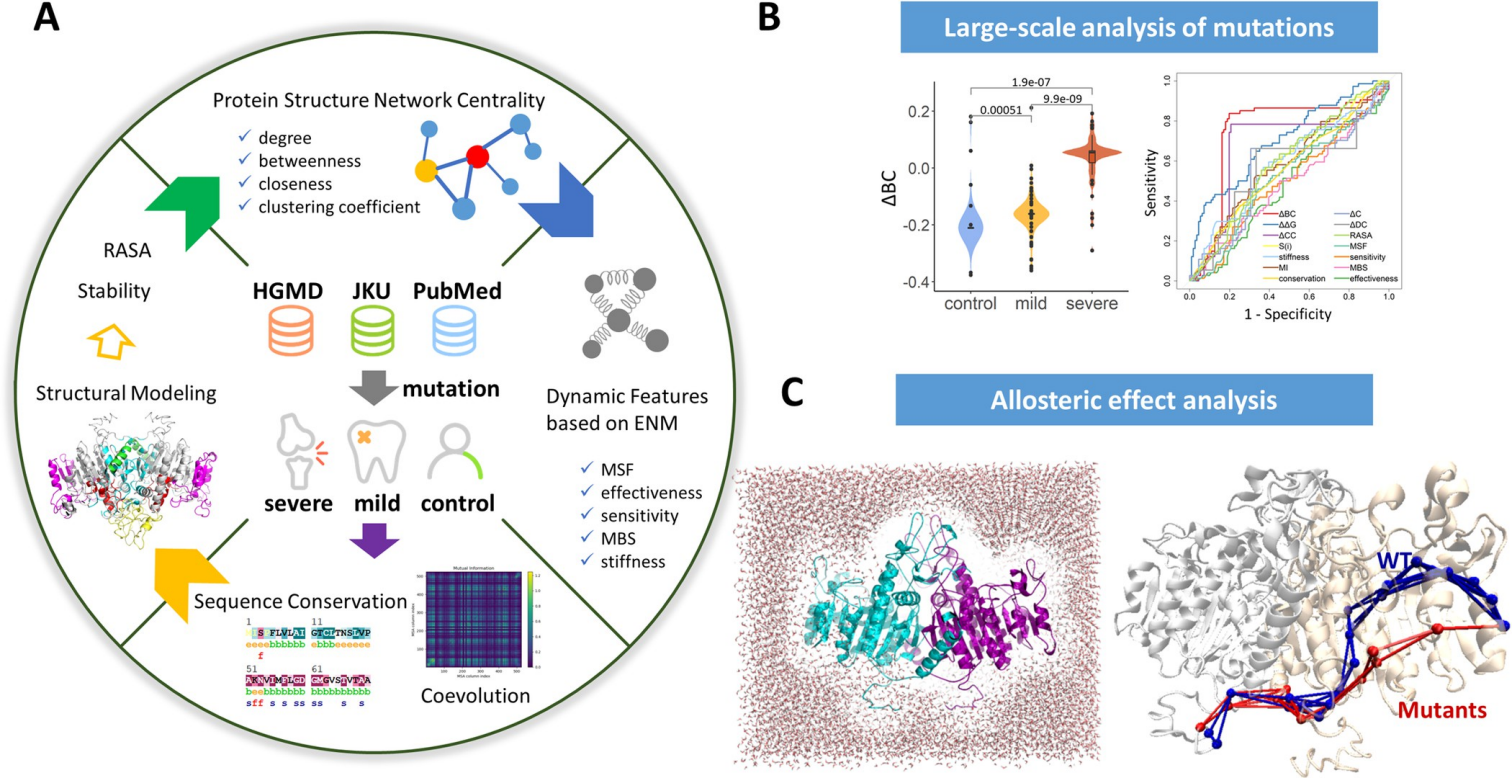
The computational workflow. (A) Beginning with the collection of *ALPL* mutations associated with different HPP phenotypes and the computational modeling of TNSALP protein structure, molecular signatures of mutational hotspots were calculated. In addition to normally used signatures, three levels of parameters for describing mutations were analyzed: conservation and coevolution analysis at the sequence level, PSN-based network matrices at the structural level, and ENM-related features at the dynamics level. (B) The classification and prediction of pathogenicity of *ALPL* mutations based on the statistical analysis of molecular signatures and the construction of machine learning models. (C) Allosteric effect analysis of predicted mutations by single-residue perturbation, molecular dynamics and long-range pathway analysis.

Our recent works have highlighted that biophysics-based and data-driven approaches, including genomic analysis, coevolution and network-based modeling provide an array of powerful tools to study disease mutations in genomic medicine and allosteric interactions [48–51]. In this work, based on this concept, we propose an integrated computational approach for the large-scale analysis of disease mutations. We collected diverse *ALPL* single-point mutations associated with neutral, mild, and severe phenotypes from various scientific gene mutation databases; the computational pipeline consisted of three steps (Fig 1). First, by performing three levels of analysis, including conservation and coevolution analysis based on the sequence level, structural modeling, energetic analysis, protein structure network (PSN) modeling, and ENM-based dynamics analysis, different molecular and network signatures were determined and attributed to three types of mutations. Second, statistical analysis including creation of a random forest model, was performed to test which molecular signatures can serve as robust predictors for classifying the three kinds of mutations. Finally, through integration of long-range perturbation dynamics and network-based approaches, we quantified the allosteric potential of selected mutation residues. Our study characterizes the mutational landscape of *ALPL* through modeling and analysis of molecular signatures and allosteric effects of mutations [52], providing new insights into the genotype-phenotype interrelationship in HPP [53].

## Materials and methods

### Data collection of ALPL mutations

With the goal of classifying various *ALPL* variants according to different phenotypes, 242 single-point loss of function variants were selected in HPP patients with mild and severe phenotypes and the control group from the *ALPL* gene mutation database (https://alplmutationdatabase.jku.at, accessed on 11 January 2021), the Human Gene Mutation Database (http://www.hgmd.cf.ac.uk/ac/index.php), the Locus Specific Mutation Databases (http://www.hgmd.cf.ac.uk/docs/oth_mut.html), and literature in the PubMed database. The collated data set of *ALPL* mutations associated with mild, severe HPP phenotypes and putative functionally non-pathogenic variants, as well as their molecular signatures, are listed in S1 Table.

### Sequence conservation and coevolution analyses

By using the Consurf server,[54] the conservation scale for each residue in the TNSALP protein was calculated (range from 1 through 9, where 1 denotes the node least conserved and 9 denotes the most conserved sites). The refined multiple sequence alignment (MSA) of the TNSALP protein can also be retrieved from the Consurf server for the following up Shannon information entropy ***S***(*i*) and mutual information (***MI***) calculations. *S*(*i*) measures the variability of specific sites of protein sequences, which is calculated as

$$S(i)=-\sum_{ai=1}^{20}P(ai)logP(ai)$$ (1)

where *P*(*ai*) is the probability of occurrence of amino acid type in the *i*^*th*^ column. *S*(*i*) varies in the range 0≤*S*(*i*)≤3.0, and a lower *S*(*i*) implies higher evolutionary conservation. Similarly, *MI* was applied as a measure of the degree of intra-molecular coevolution between residues. The *MI* associated with the *i*^*th*^ and *j*^*th*^ sequence positions is defined as a *N*×*N* matrix of the form

$$I(i,j)=\sum_{xi=1}^{21}\sum_{yj=1}^{21}P(xi,yj)log\frac{P(xi,yj)}{P(xi)P(yj)}$$ (2)

where P(xi, yj) is the joint probability of observing amino acid types *x* and *y* at the respective sequence positions, *i* and *j*; P(xi) is the marginal/singlet probability of amino acid of type *x* at the i^th^ position. Gaps are counted as residue type 21.[55] I(i, j) varies in the range [0, I_max_], corresponding to fully uncorrelated and most correlated pairs of residues. The coevolution of a mutation was measured by the average *MI* values corresponding to each residue. The calculation of *S*(*i*) and *MI* was performed by Evol [56].

### Structural modeling and protein stability analysis

A protein homology model for human TNSALP was constructed by using the MODELLER V9.19 [57] platform, using a template corresponding to the human placental alkaline phosphatase (PDB id: 1ZED [58]) and a very slow refinement. The template had a sequence identity of 57% to TNSALP and an X-ray crystal structure resolution of 1.57 Å. The quality of the modeled structure was evaluated by Verify 3D [59], PROCHECK,[60] ProSA,[61] and ERRAT [62]. Each single *ALPL* missense variant and its mutant structures were automatically generated by FoldX [63] and its effect on protein stability can be measured by the difference folding Gibbs free energy (***ΔΔG*** values) between the wild type (WT) and the mutated forms of TNSALPs. The accessible surface area (ASA) is the atomic surface area of a protein that is accessible to a solvent, and the relative ASA (***RASA***) attribute is the per-residue ratio between the calculated ASA and ’standard’ ASA for a particular mutational residue. As a measure of amino acid side-chain accessibility, the RASA also serves as a quantitative predictor of variant pathogenicity [64]. The RASA calculation of TNSALP was performed using through PSAIA 1.0. [65]

### Amino acid contact energy network analysis

Amino Acid Contact Energy Networks (AACENs) for the TNSALPs were constructed and analyzed by using the NACEN R package based on static structures [66]. A node in the network denotes a single amino acid residue, and edges are defined by the environment-dependent residue contact energy between two nodes [67,68].


$$e_{ij}=-ln\;(N_{ij}N_{00}C_{i0}C_{j0}/N_{i0}N_{j0}C_{ij}C_{00})$$ (3)

Based on AACENs, some network centralities have been defined.[69] The simplest centrality measure is the degree centrality (***DC***) of a node *i* in AACENs, defined as the total number of nodes to which it is directly connected to. The betweenness centrality (***BC***) was defined as the number of times residue *i* was included in the shortest path between each pair of residues in the protein, normalized by the total number of pairs. It is calculated by

$$BC=\sum_{j,k\in N,j\neq k}\frac{n_{jk}(i)}{n_{jk}}$$ (4)

where n_jk_ is the number of shortest paths connecting *j* and *k*, while n_jk_(i) is the number of shortest paths connecting *j* and *k* and passing through *i*. The closeness centrality (***CC)*** for a node was calculated by the reciprocal of the average shortest path length, which can be calculated as follows:

$$CC=\frac{\left(n-1\right)}{\sum_{k\in N,k\neq m}L(m,k)}$$ (5)

where N is the set of all modes and *n* is the number of nodes in the network. The clustering coefficient (***C***) measures the degree to which nodes tend to cluster together and is defined as:

$$C=\frac{2e_{i}}{K_{i}(K_{i}-1)}$$ (6)

where *K*_*i*_ is the degree of node *i* and *e*_*i*_ is the number of connected pairs between all neighbors of *i*.

### Dynamic features based on elastic network models

In ENM approaches [35,36], each node represents a Cα atom in proteins and each edge is a spring γ for connecting two sites within a given cutoff distance r_c_. Two most commonly used ENM methods, the GNM and the ANM, are adapted in this paper. The total potential energy of the ANM and GNM systems with N nodes are expressed as

$$V_{GNM}=-\frac{\gamma }{2}[\sum_{i=1}^{N-1}\sum_{j=i+1}^{N}(R_{ij}-R_{ij}^{0})∙(R_{ij}-R_{ij}^{0})\Gamma_{ij}]$$ (7)

$$V_{ANM}=-\frac{\gamma }{2}[\sum_{i=1}^{N-1}\sum_{j=i+1}^{N}{\left(R_{ij}-R_{ij}^{0}\right)}^{2}\Gamma_{ij}]$$ (8)

where R_ij_ and $R_{ij}^{0}$ are the instantaneous and equilibrium distances between nodes *i* and *j*, and Γ_*ij*_ is the *ij*^th^ element of the N×N Kirchoff matrix Γ, which is written as

$$\Gamma_{ij}=\left\{ \begin{array}{c} -1\;i\neq j,R_{ij}\leq r_{c}\\ 0\;i\neq j,R_{ij}>r_{c}\\ -\sum_{i,i\neq j}\;\Gamma_{ij}\;i=j \end{array}\right.$$ (9)

In our study, r_c_ between protein nodes were 7 Å and 13 Å for GNM and ANM, respectively. In comparison with GNM, which only measures fluctuation, ANM provides additional information on the motion directions of each residue. Herein, the GNM and ANM calculations were performed by ProDy [70].

The normal modes are extracted by eigenvalue decomposition Γ = U∧U^T^. U is the orthogonal matrix whose k^th^ column U_k_ is the k^th^ mode eigenvector, and ∧ is the diagonal matrix of eigenvalues, λ_k_. Mean-square fluctuations (***MSF***) of a residue are given by

$$\langle {\left(△R_{i}\right)}^{2}\rangle =\frac{3k_{B}T}{\gamma }\sum_{k}{[(U}_{k}\Lambda {U_{k}^{T})}^{-1}{]}_{ii}$$ (10)

where k_B_ and T represent the Boltzmann constant and temperature, respectively. Based on ENM calculations, several other kinds of matrices can be generated. Form the perturbation response scanning (PRS) matrix [71], two dynamic features of ***effectiveness*** and ***sensitivity*** were defined as row and column averages of the matrix, respectively. The effector residues most effectively propagate signals in response to external perturbations. The sensor residues can easily sense signals and respond with dynamic changes. Directly from the Kirchoff matrix, its eigenvalues can be used to ascertain how important each node is to maintain the overall mechanical connectedness of the network. This amounts to measuring how much the network Laplacian spectrum changes when the connections, or couplings, of a node with its neighbors are deleted. As such, the mechanical bridging score (***MBS***) for a given mutation reflects the response ability of this residue. In the symmetrical stiffness matrix, the elements describe the effective spring constants associated with each residue pair. The ***stiffness*** for individual mutational residues is obtained by averaging all the elements in the corresponding row/column of the matrix. A detailed description of these dynamic features can be found in Bahar’s work [38]. The statistical analysis of all features was performed in the R language (https://www.r-proj ect.org/, R Core Team, 2017), version 3.6.2. Plots were generated using the R package ggplot2, version 3.3.3 [72]. Statistical significance was determined by the Wilcoxon signed ranked test (P <0.01) in our analysis.

### Molecular dynamics simulations

MD simulations were performed on WT and mutant TNSALPs for the conformational dynamics study. The minimized structures were then slowly heated from 0 to 303.15 K over 100 ps and a two-step equilibration (each 100 ps) was carried out to ensure the correct temperature and pressure of the solvated system was attained. Firstly, the temperature was set at 303.15 K (NVT—constant number of particles, volume and temperature) using the V-rescale thermostat. Subsequently, equilibration at 1 atm (NPT—constant number of particles, pressure and temperature) was performed using the Parrinello-Rahman barostat [73]. The equilibrated systems were subjected to production MD for 500 ns using the GPU-enabled version of the GROMACS software package (version 5.1.4)[74] with the AMBER99SB-ILDN force field [75], using a integration step of 2 fs. The structures were immersed in an octahedral box filled with TIP3P water molecules, imposing a minimum distance of 15 Å between the solute and the box. The nonbonded interaction potential was smoothly switched off between 10 and 12 Å, beyond which coulombic interactions were treated with the particle-mesh Ewald method [76]. The LINCS algorithm [77] was used to constrain the hydrogen containing bonds. The atomic positions were saved every 50000 steps (100 ps) for analyses. For each system (WT and mutant), three independent replicas of 500 ns were performed. GROMACS analysis toolkit utilities were used to analyze MD trajectories produced during the last 400 ns production run, and root mean square deviations (RMSDs) and root mean square fluctuations (RMSFs) were calculated for each system.

### Allosteric mutation analysis

Two methods were used to investigate the allosteric effects of mutations. The dynamic residue network analysis (DRN) was performed by the program, MD-TASK [78]. For each protein system, the network was constructed by extracting C_α_ and C_β_ atoms of MD trajectories as nodes, and the edge was created when two nodes were within 6.5 Å. The network measure of BC was also calculated based on DRNs. Then, the allosteric communication pathways were calculated by connecting mutational sites and allosteric sites with the shortest edges, using the Floyd-Warshall algorithm [79]. The AlloSigMA server [80,81] was used to evaluate the allosteric effects of each mutation based on the structure-based statistical mechanical model of allostery. This statistical mechanical model estimates the allosteric free energy difference ***Δg*** of each residue to perform the allosteric signaling and mutation analysis in TNSALP.

## Results

### Sequence and structural landscapes of ALPL mutations

**Fig 2:**
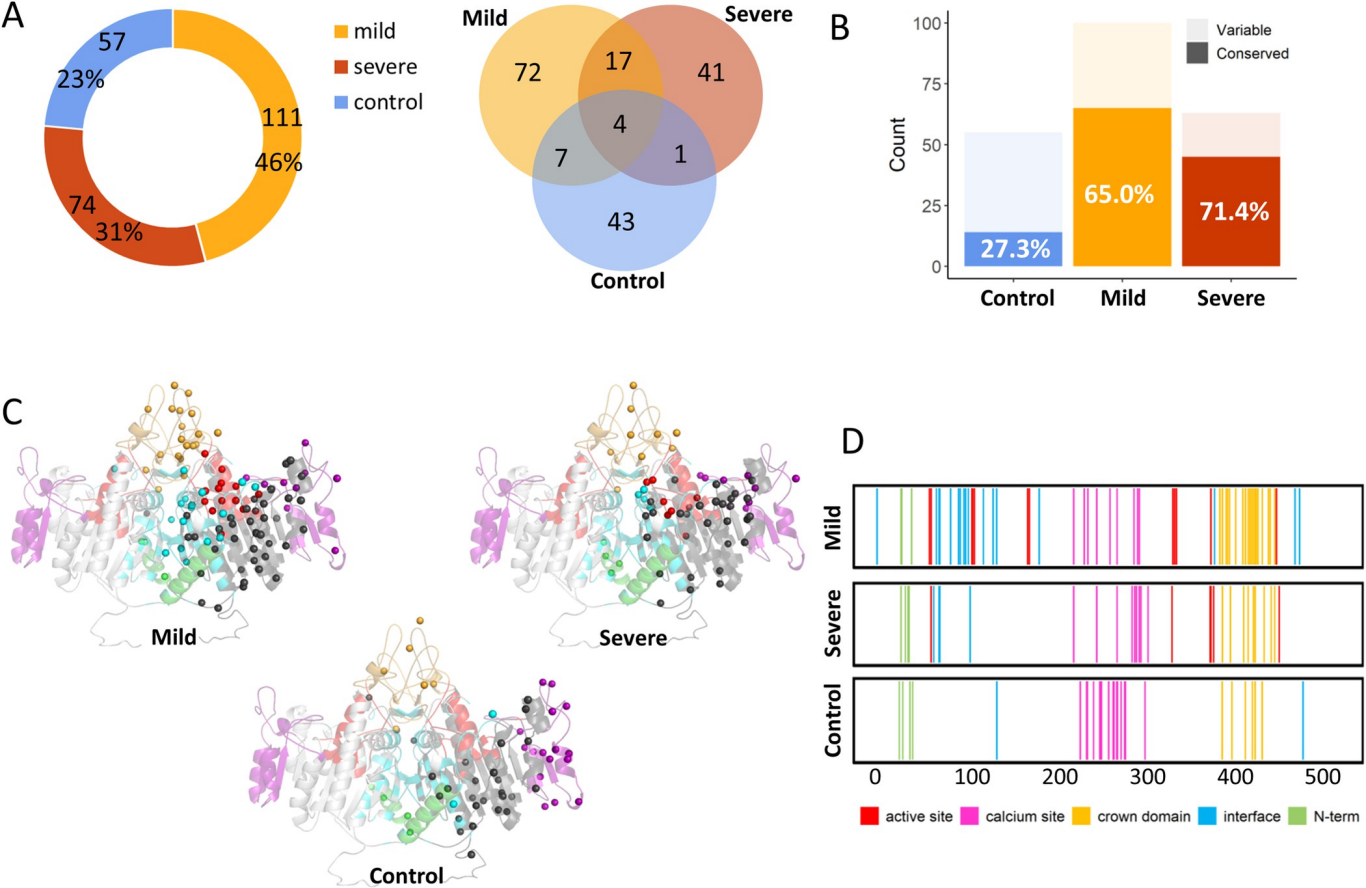
Sequence and structural analysis of *ALPL* mutations in the TNSALP protein. (A) The composition and statistics of *ALPL* mutations of based on the three categories: mild HPP, severe HPP, and control group. (B) The conservation distribution of mutations related to different phenotypes. (C) The structure of the TNSALP protein and the distribution of mutation sites in the active site (red), calcium site (purple), crown domain (brown), dimeric interface (blue), and N-terminal domain (green). Missense mutations in the three groups are shown as colored spheres based on the coloring scheme of the domains to which they belong. (D) Distribution of different clinical phenotypic mutations across the length of the TNSALP protein.

To clarify the sequence characteristics of genotype and phenotype underlying *ALPL*, we first divided all mutations into three categories according to the severity of HPP: the mild form, severe form, and control group. Fig 2A shows the distribution of mutational data, while the detail description can be found in S1 Text. The primary sequence analysis showed that there were 261 conserved residues (conservation score > 5, see S2 Fig) among the 524 full length residues of the TNSALP monomer. Among the mutation sites that cause severe phenotypes, more than 71.0% of the sites are highly conserved, while in the mild group, this proportion is 65.0%, and in the control group, it is much lower, at only 27.3% (Fig 2B). It is worth noting that half of these highly conserved sites in the control group are from the intersection of the mild group and the severe group. Furthermore, the evolutionary landscape of the three types of *ALPL* mutations was plotted in terms of ***S***(*i*) and ***MI*** profiles.

**Fig 3:**
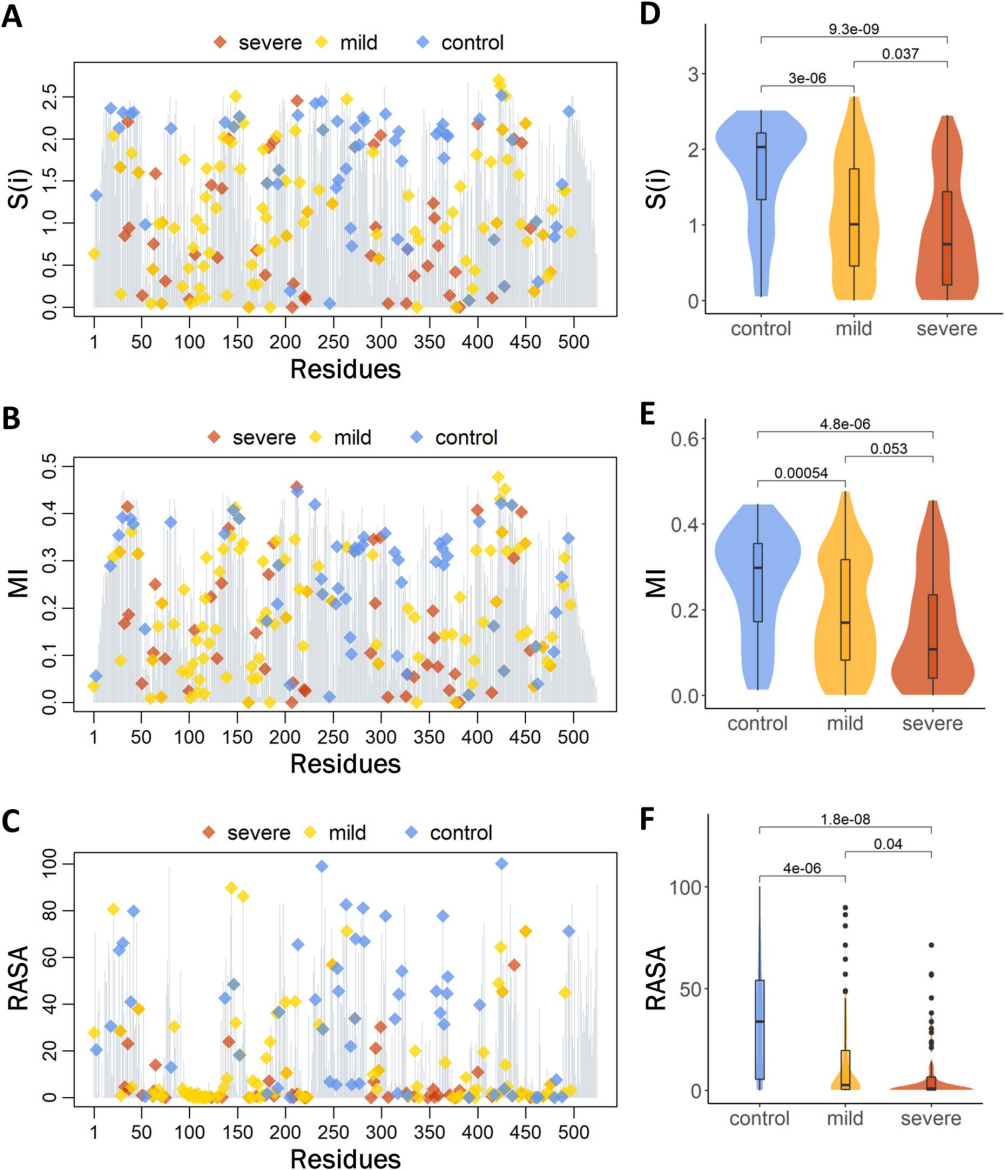
The (A) entropy (S(i)), (B) coevolutionary (*MI*) and (C) relative solvent accessible area (RASA) profiles for each residue in TNSALP; mild, severe, and control mutations are highlighted as yellow, red, and blue diamonds, respectively. The comparison of (D) the S(i), (E) *MI* and (F) RASA, between the control group mutations and mild and severe mutations. Statistical significance was determined by the Wilcoxon signed-ranked test, with *P* values<0.01.

In general, disease-causing variants were more likely to occur at conserved sites, while the non-pathogenic variants are more likely to occur at the less conservative sites (Fig 3A). The distributions of ***S***(*i*) and ***MI*** scores for mutations were very similar, and a significant positive correlation between coevolution and entropy was observed (S3A Fig), and the distributions of ***S***(*i*) and ***MI*** scores for mutations were also very similar. In contract with ***S***(*i*), the higher ***MI*** values of amino acids correspond to residues that were highly coevolutionary. As shown in Fig 3B, pathogenic mutations including both severe and mild mutations occurred at lower coevolutionary residues.

The 3D structure of TNSALP was then computationally modeled by using the crystal structure of human placental alkaline phosphatase as the template (S4 Fig and S1 Text). By mapping all the mutations onto the modeling TNSALP structure, we identified the spatial distribution of the three kinds of mutations (Fig 2C and 2D). The major difference in the spatial distribution between pathogenic and control groups was that the control group had no mutations at the active site, thereby suggesting the role of active sites in HPP pathogenicity. According to the RASA (Fig 3C), residues were classified into three classes, i.e., buried areas (RASA < 5%), semi-exposed areas (between 5% and 30%), and fully exposed areas (RASA > 30%). Although different types of mutations were distributed across all functional domains, disease-causing variants were more likely to occur in buried areas (122 mutations, 65.9%). In contrast, non-pathogenic variants were more likely to occur at the surface (32 mutations, 56.1%).

The statistical analysis of ***S***(*i*), ***MI*** and RASA for control, mild, and severe mutations showed statistical significance between the control and mild, and control and severe mutations; however, there was no statistical significance between the mild and severe mutations (Fig 3D–3F). Accordingly, structural environment and evolutionary studies have found that the HPP-causing *ALPL* mutations, especially severe mutations, are located in conserved, less coevolutionary, and buried residues with small ***S***(*i*), ***MI*** and RASA scores.

### Protein stability effects of severe and mild mutations: Structural determinants of TNSALP stability and energetic hotspots

**Fig 4:**
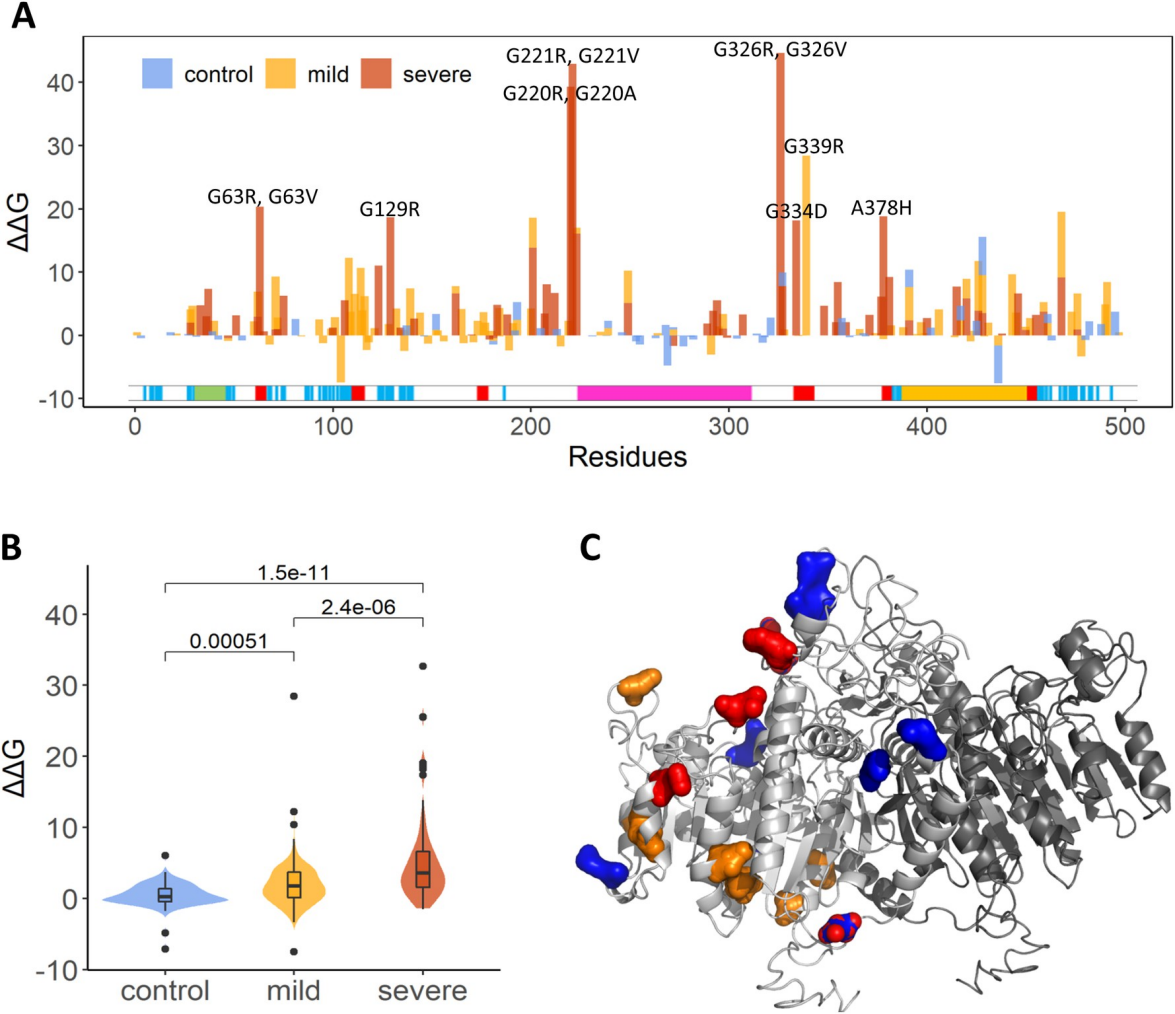
(A) The folding free energy changes induced by single point mutations. The profiles of mild, severe, and control mutations are shown as yellow, red and blue bars, respectively. (B) The significant difference between the predicted folding free energy change of *ALPL* single residue variations in the control, mild, severe groups was measured by Wilcoxon. test, with *P* values<0.01. (C) The structural distribution of disease-causing mutations with low ΔΔG.

To explore the thermodynamic determinants of mutational hotspots and evaluate their functional significance, FoldX approach [63] was adopted for the predicting of the free energy changes induced by single point mutations. As shown in Fig 4A, we found that most of the disease mutations were associated with the increase in the folding free energy (ΔΔG>0), thus leading to the reduced protein stability. Importantly, the largest destabilization changes upon substitutions correspond to the group of mutations with severe phenotype (Fig 4A). At the same time, the mean value of ΔΔG for most mutations in the control group, were smaller than 1.0 kcal/mol. Furthermore, as shown in Fig 4B, the predicted folding free energy changes ΔΔG for mutations that cause a severe phenotype were significantly larger than that the ones in the mild group and in the control group. These findings indicated that most of the disease-causing mutations are associated with a significant decrease in the protein stability, and this trend varies in different phenotypes.

In addition, ΔΔG provides a benchmark or metric that can be used to compare with physicochemical properties for large-scale analysis of other mutations. We have compared the ΔΔG with the RASA, ***S***(*i*) and ***MI***, and weak correlations were found among them (S3B–S3D Fig). The low coefficients imply that the three properties provide additional measures of ΔΔG for describing *ALPL* mutations, namely pathogenic mutations tend to have a high ΔΔG value and low RASA, ***S***(*i*), and ***MI*** values. For example, pathogenic mutations, including G220R, G221R, G326R, G129R, P108L, R223W, and G63R (severe mutations), and G339R and A468V (mild mutations) showed obvious energy changes but with low RASA, ***S***(*i*), and ***MI*** scores. Based on the above analysis, we concluded that most of the “severe” and “mild” mutations were predicted to have large ΔΔG values and differed in terms of evolutionary conservation and physicochemical properties.

It is worth noting, however, that severe mutations R272L, N47I, L299P, R272H, H438L, and R450C, and mild mutations R272H, R450H, V424A, K264R, E21K, V424M, R184Q, E84V, N47I, K422R, and T148I showed a relatively moderate free energy change (Fig 4). At the same time, some non-pathogenic mutations I269V, H482N, F327C, A488S, T277A, T255I, I317L, and D239Y, displayed larger free energy changes. Structurally, these mutations were distributed in different domains and did not cluster together (Fig 4C). These results indicated that functional effects of pathogenic mutations may be determined not only by the local energetic changes but could be also influenced by changes in the long-range allosteric interactions and potential reorganization in the global interaction networks.

### Modeling of residue interaction networks reveals allosteric signatures of *ALPL* mutations

**Fig 5:**
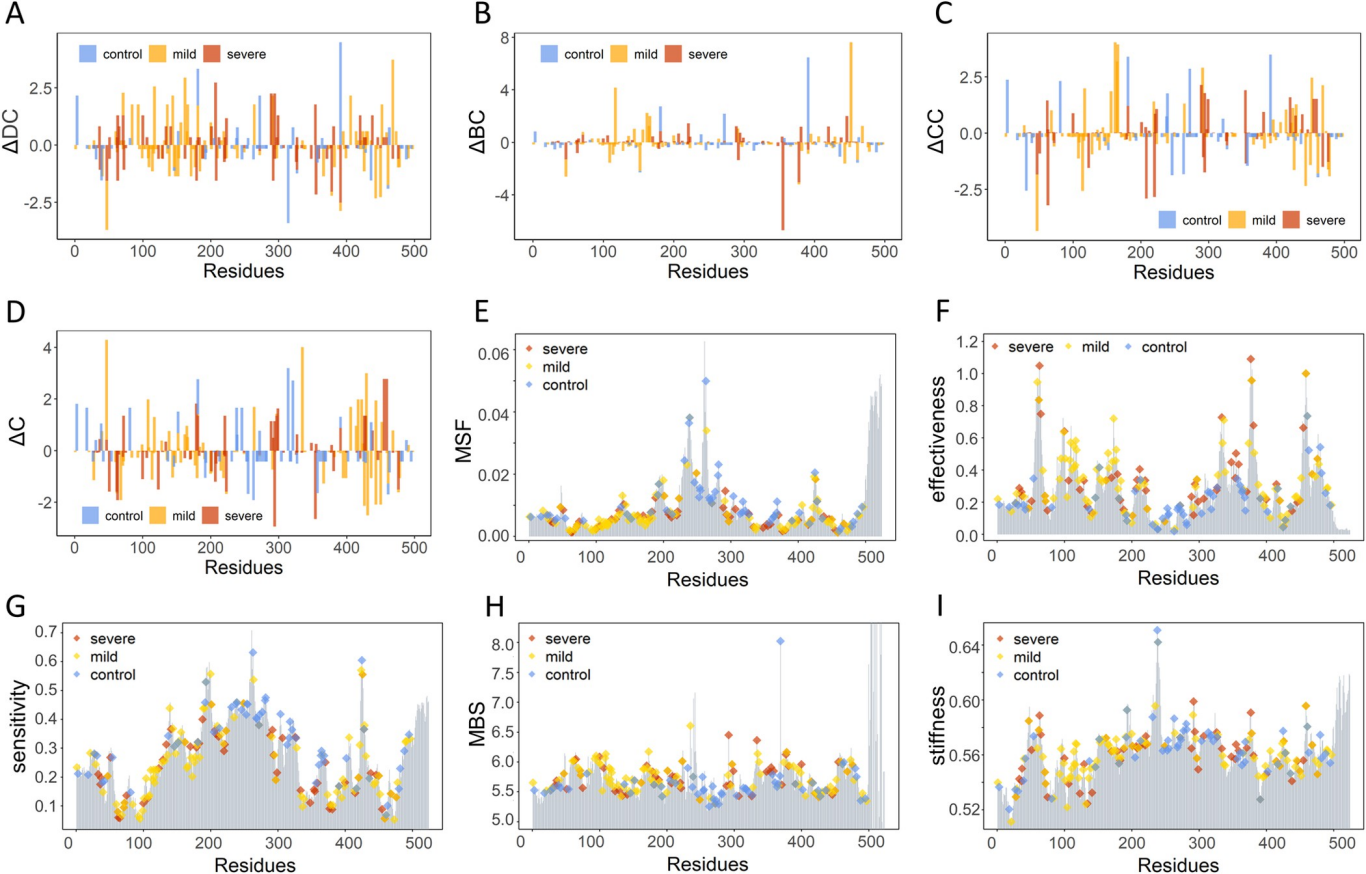
Network topological and dynamic analysis of mutations in *ALPL*. (A)ΔDC, (B) ΔBC, (C) ΔCC, (D) ΔC, (E) MSF, (F) effectiveness, (G) sensitivity, (H) MBS, and (I) stiffness profiles for the three types of mutations. Control, mild and severe mutations are represented as blue, orange, and red bars, respectively.

**Fig 6:**
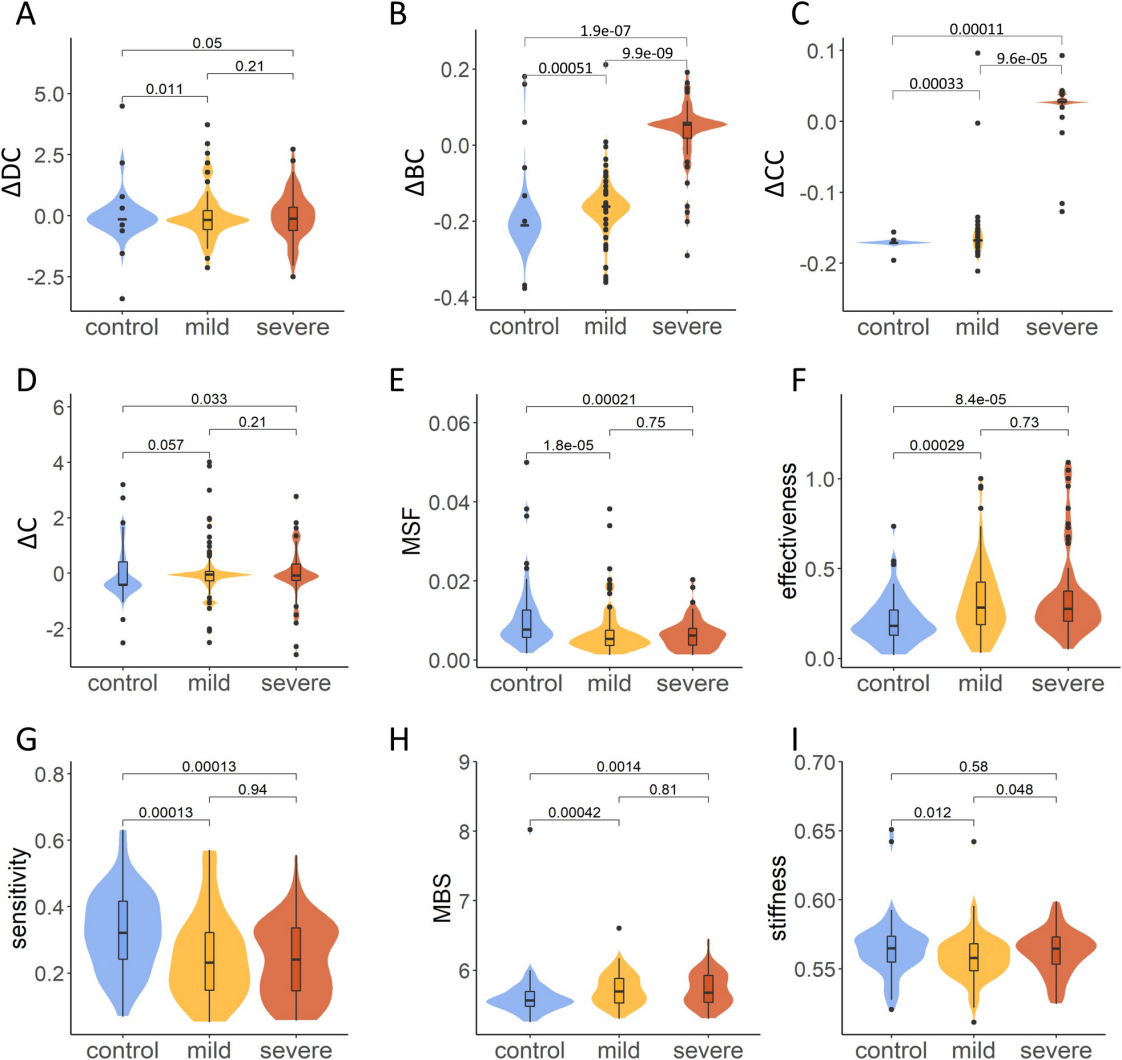
Comparison of the change of four network centralities and five dynamics-based parameters, including (A)ΔDC, (B) ΔBC, (C) ΔCC, (D) ΔC, (E) MSF, (F) effectiveness, (G) sensitivity, (H) MBS, and (I) stiffness, among mutations in the control, mild, and severe groups by Wilcoxon signed ranked test.

Network theory has become a widely used method to quantify protein structures and functions in terms of their topological connectivity [82]. In our analysis, we used the difference between network centrality measures of mutant and WT values to describe the topological change of TNSALP AACENs caused by *ALPL* mutations. For each amino acid substitution, four common network parameters including DC, BC, CC, and C were calculated (see Materials and Methods for a detailed definition of these parameters). The mutation-induced changes ΔDC, ΔBC, ΔCC, and ΔC were computed on each mutation and analyzed for the mild, severe, and control groups, as illustrated in Figs 5 and 6.

The profiles in Fig 5A–5D indicted that most of the mutations can induce a wide range of changes in the values of DC, CC, and C network parameters. In general, some common peaks were found in the ΔDC, ΔBC, and ΔCC profiles, such as the severe mutation R391H and three mild mutations G162S, T166I, and R272C. Detailed analysis of the ΔDC profile showed that its peaks corresponded to pathogenic mutations, including severe mutations R391C, Q207P, D294A, and L289F and mild mutations A468V, G162S, Y117C, G144E, E291K, and D406G. These significant peaks aligned precisely with functional centers, including hotspot positions involved in the crown domain, the homodimer interface, and the Ca^2+^ binding site, thereby suggesting their latent functional roles. In comparison, smaller variations were observed in the ΔBC profile (Fig 5B), but much sharper peaks for severe mutations F355I, D378H and A446T and mild mutations E452K, Y117C, G162S, R152C, and T166I were found. Functional domain analysis revealed that E452 and D378, R391, and A446, R272 are involved in the active site, crown domain, and Ca^2+^ binding site, respectively. Eleven peaks were found in both the ΔCC and ΔC profiles. In the ΔCC profile (Fig 5C), eight corresponded to pathogenic mutations, including G162S, T166I, G63R, T165I, L208F, E291K, G220R, and N47I, and only one (E291K) was located at the functional domain. In the ΔC profile (Fig 5D), the other set of pathogenic mutations, peaks corresponded to R335T, N47I, E429K, D294Y, G455D, V459F, E354D and V431A, including many functional sites. Among these, R335T and G455D were located at the active site, D294Y at the Ca^2+^ binding site, and E429K at the crown domain.

A series of ENM-based dynamics descriptors, including GNM-based MSF, PRS-based effectiveness and sensitivities, and ANM-based mechanical bridging score (MBS) and stiffness, have been introduced to enhance the predictive ability of disease-related mutations [38]. Here, we employed these dynamic features to systematically characterize different types of *ALPL* mutational sites. First, mapping the three types of mutations onto the dynamic profiles based on the TNSALP protein provided primary insight into the interpretation of the functional impact of variants in light of the intrinsic dynamics of the mutational site (Fig 5E–5I). As shown in the MSF profile (Fig 5E), pathogenic mutations including both mild and severe mutational sites always demonstrated lower MSF values, except for some pathogenic mutations at the Ca^2+^ binding domain. Some particular pathogenic mutations, located at minimal positions that correspond to hinge sites have been found, such as M62, G63, S65, A377, D378, and H381, which belong to severe mutational sites, and D60, V461, and G473, which correspond to mild mutational sites. Structurally, these hinge mutational hotspots are located at the active site and the dimer interface. These conformational dynamic signatures of disease-associated mutations have been revealed both at the genome [83] and proteome-levels [33].

Regarding the PRS analysis, two matrices were used to quantify the allosteric effect of each residue, which are effectiveness and sensitivity, for evaluating the propensity of residues to act as sensors or as effectors of allosteric signals. As shown in the profiles in Fig 5F and 5G, the distribution of effectiveness and sensitivity for *ALPL* mutation hotspots shows different trends. In general, most of the pathogenic mutations match almost exactly with some peaks of the effectiveness profile, with sharp dominant peaks being found for three severe mutational sites (G63, A377, and V459). On the other hand, the peaks of the sensitivity profile correspond to some control mutational sites, while pathogenic mutational sites have smaller sensitivity, such as V95 and G473 in the mild group and G63, S65, and V459 in the severe group. As two unusual ENM-based dynamic features, the distribution of mutations in MBS and stiffness profiles are shown in Fig 5H and 5I. For the MBS profile, the overall distribution of pathogenic mutational sites is larger than that of control mutational sites, highlighting the importance of pathogenic mutational sites in maintaining the stability of the *ALPL* protein.

In addition, both ΔBC and ΔCC profiles showed significant differences between groups of mutations (Fig 6). As shown in Fig 6B, the mean values of ΔBC for the severe, mild, and control groups were 0.05, 0.16 and 0.21, respectively, with *P* = 1.889e-07, 9.906e-09, and 0.0005129, respectively, by the Wilcoxon signed rank test between the severe and mild, severe and control, and mild and control groups. In contrast, as shown in Fig 6C, the mean values of ΔCC for the severe, mild, and control groups were 0.03, 0.17 and 0.17, with *P* = 9.646e-05, 0.0001094, and 0.0003274, respectively, by the Wilcoxon signed rank test, between the severe and mild, severe and control, and mild and control groups.

An important revelation of the protein network centrality analysis is that pathogenic *ALPL* mutations typically lead to significantly higher variations in ΔBC and ΔCC parameters as compared to the control group (Fig 6). Given topological nature of the network parameters measuring the global effect of mutations, the observed magnitude of the mutation-induced changes in the betweenness and closeness centrality suggests that pathogenic mutations, and especially severe mutations, may significantly alter the long-range connectivity of the interaction network. In particular, the ΔBC profile displayed the most pronounced changes and highlighted the potential allosteric effects for pathogenic mutations [84].

To clearly observe the ability of dynamic parameters to distinguish mutations in different groups, statistical analysis was further performed on the data of severe, mild, and control mutational sites. As shown in Fig 6E, the control group had the highest mean MSF, with *P* = 1.8e-05 and 0.00021 by the Wilcoxon signed rank test, in comparison with mild and severe mutations, showing significant differences between both pathogenic mutational sites and the control group. However, significant differences were not found between mild and severe mutational sites, with *P* = 0.75. Similar significant results were also found for effectiveness (Fig 6F), sensitivity (Fig 6G), and MBS (Fig 6H), while significant differences in stiffness could not be found among the three groups of mutations (*P*> 0.01, Fig 6I).

### Atomistic simulations and dynamic network modeling determine the effects of severe phenotype-associated mutations on allosteric communications

**Fig 7:**
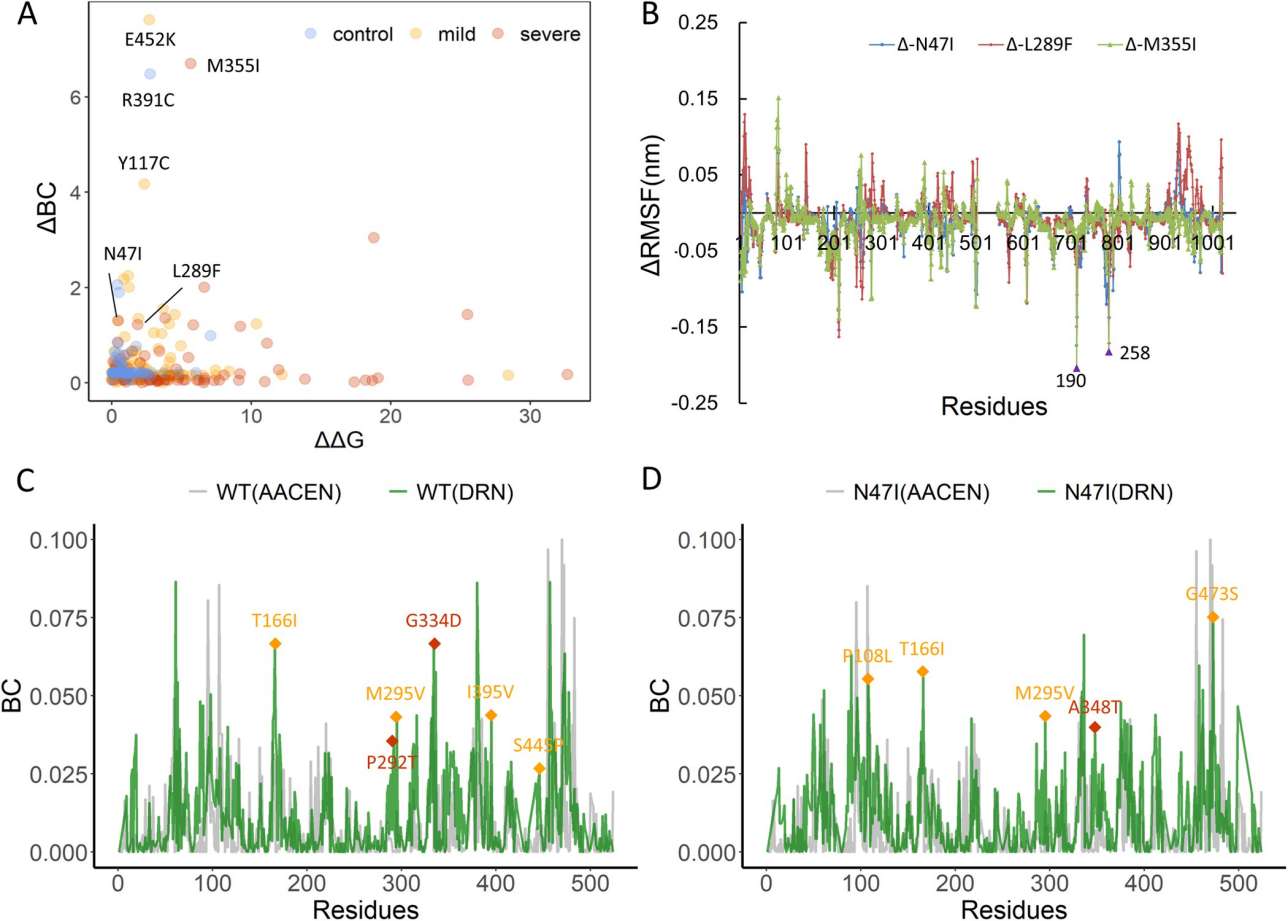
(A) The distribution of ΔΔG and ΔBC for *ALPL* mutations. Scatterplot showing the distribution of ΔBC vs ΔΔG of different mutation types. Severe, mild and control mutations are depicted in red, yellow, and blue, respectively. N47I, L289F, and M355I are three severe mutations with low ΔΔG and high ΔBC. Among the three significant mutations predicted by the scatterplot, two (E452K and R391K) were not originally included in the severe mutation group but were validated as two severe mutations in the newly collected clinical samples. (B) Mean values of three replicas of the differential RMSF (ΔRMSF) of N47I (blue), L289F (red), and M355I (green) with respect to WT. For each system, a replica of 500 ns was singled out to compare the BC values of the two different networks of TNSALP WT (C) and N47I (D) mutant. The green and grey lines show the BC values of residues of DRN and AACEN. Mild and severe mutations are highlighted as yellow and red diamonds, respectively.

Based on previous results, some severe phenotype-related mutations have relatively low ΔΔG but higher ΔBC values in network topology. To gain energetic and topological insights, we compared ΔBC and ΔΔG values for all mutations. The scatterplot revealed that N47I, L289F and M355I were three severe mutations with large ΔBC but low ΔΔG (Fig 7A). To determine the dynamic effects induced by severe phenotype-associated mutations, we performed a set of three independent replicas, all-atom MD simulations for WT and three mutant TNSALPs caused by N47I, L289F, and M355I variants (Fig 7). The RMSD values of Cα atoms served as an overall measurement of the departure of the structures from the initial coordinates (S5A–S5D Fig). All the systems became convergent for duration of 100–500 ns, and the RMSD values became stable during the last 400 ns of the simulations. The conformational dynamics results (S5E–S5H Fig) revealed that the profile of RMSF was very consistent in three replicas of each system and also consistent between the WT and mutant systems, and no conformational change occurred at the active site, further explaining why ΔΔG for these severe mutations was small. The differential fluctuation ΔRMSF, with respect to WT, showed considerable changes in regions around the ion binding pocket and the Ca^2+^ binding site located in the other monomer, with two of the largest peaks at residues N190 and S258 (Fig 7B). This suggests the existence of allosteric communication between the mutant sites and these two regions. Accordingly, in the four systems, we selected mutant sites in chain *A* as starting points and N190 and S258 of chain *B* as the target residues to further identify specific allosteric pathways caused by severe phenotype-associated mutations. In addition, the comparisons of BC values based on DRNs among three independent MD replicas (S6 Fig), as well as between AACENs and DRN (Figs 7C and 7D and S7) were performed. The overall similar profiles of BC not only show the reproducibility of MD simulations, but also indicate that BC provides a robust network index. The difference is that DRN can capture more peaks that correspond to disease mutations, such as T166I, M295V, I395V, and S445F (mild mutations) and P292T and G334D (severe mutations) in WT (Fig 7C), while P108L, T166I, M295V, and G473S (mild mutations) and A348T (severe mutations) in N47I mutant (Fig 7D).

**Fig 8:**
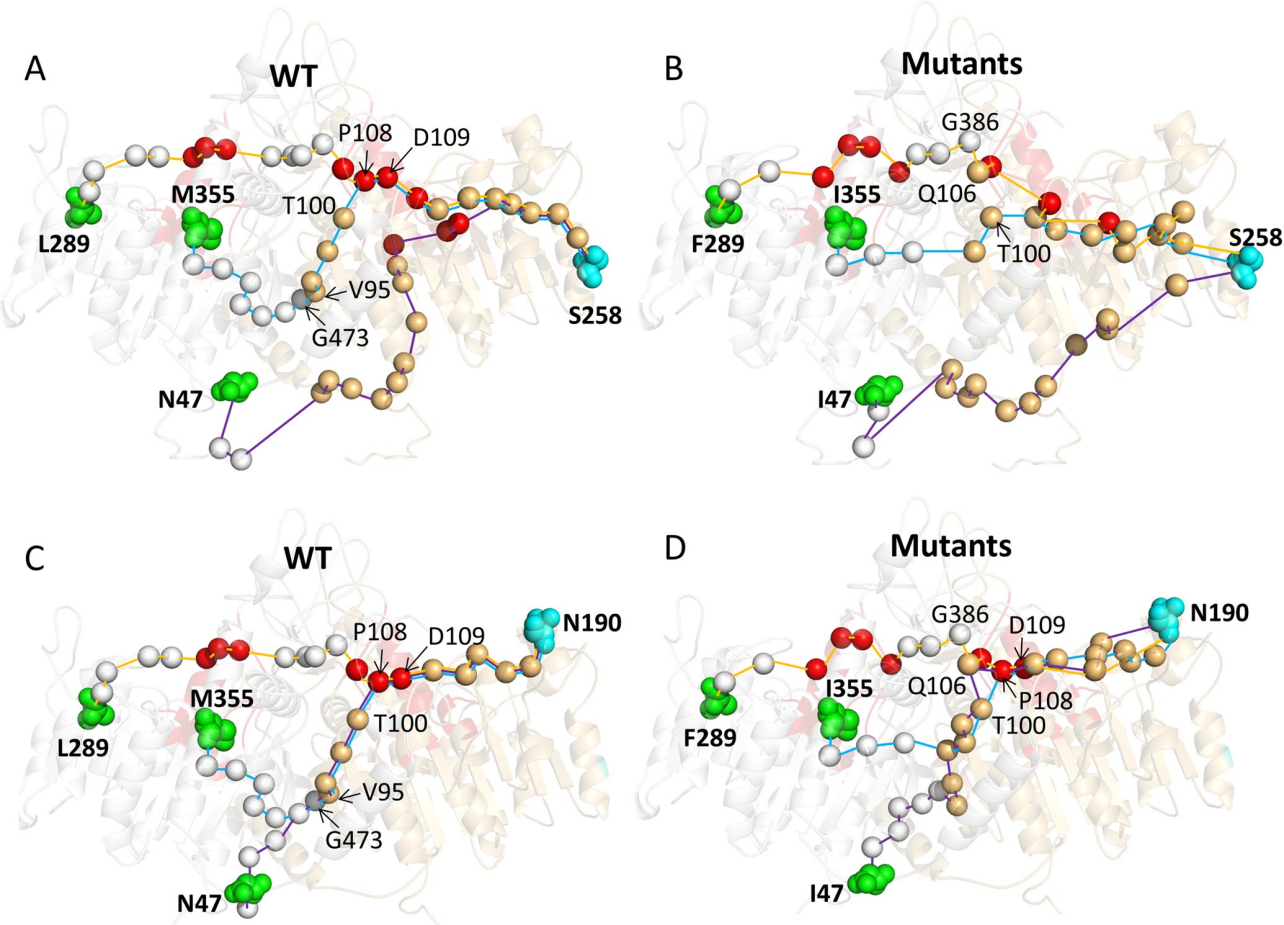
Allosteric paths originating at three mutational sites and terminating at S258 in the WT (A) and mutant states (B), as well as terminating at N190 in the WT (C) and mutant states (D), respectively. The TNSALP structure is depicted as represented by a semitransparent colored cartoon, and the starting and ending residues of all the paths are represented as green and cyan spheres, respectively. The alpha-carbon of the path through the residues is shown as silver (chain *A*) and orange (chain *B*) spheres, in which active sites are represented by red spheres.

The shortest path algorithm based on dynamic network models was employed to identify specific allosteric pathways (Table 1). As shown in Fig 8, the intermolecular interaction of *A*: G473-B: V95 was predicted as the key bridge in WT, and the other interaction of *A*: G386-B: Q106 was predicted as the key bridge in mutants. It was, therefore, determined that WT and mutant TNSALPs use different allosteric interfaces for signal transmission. Overall, pathway plasticity was found to exist, although the structures between WT and mutants were well conserved, conforming to the recent function-centric allosteric regulation study [85]. Among these pathways, a common region including three active site residues (T100→P108→D109) corresponding to mutational sites was also found, suggesting that these mutational paths may lead to the same functional state. In particular, the molecular signatures showed that T100, P108, and D109 have relatively high effectiveness and ΔBC, demonstrating that these nodes are key sites for structural signal propagation.

**Table 1:** The constituent residues of the shortest pathway from the three severe mutational sites (N47, L289, and M355 to N190/S258). Residues in the two chains are denoted by different colors.

Class	Start—end	Pathways
WT	N47 – N190	T48→M467→L470→H472→V95→A96→S98→T100→P108→D109→G126→R184→D185→Y187→S188
	L289 – N190	F290→E291→Q296→Y297→H338→G339→H340→F383→T384→F385→G386→V107→P108→D109→G126→R184→D185→Y187→S188
	M355 – N190	I359→V357→F462→S463→L471→H472→G473→V95→A96→D98→T100→P108→D109→G126→R184→D185→Y187→S188
Mutants	I47 – N190	M467→L470→L471→H472→G473→F94→V95→L97→S98→K99→V107→P108→E125→A182→D185
	F289 – N190	F290→M295→R335→G339→H340→H381→F383→T384→G386→Q106→V107→P108→E125→A182→D185
	I355 – N190	I359→V375→T176→A460→L97→S98→T100→P108→D109→G126→D183→W186→Y187→S188
WT	N47 – S258	P519→L523→K45→L46→N47→N49→V50→A51→V54→M56→F57→L58→P174→S175→I204→A205→Y206→L252→T255
	L289 – S258	F290→E291→Q296→Y297→H338→G339→H340→F383→T384→F385→G386→V107→P108→D109→A111→V128→D181→I204→A205→Y206→L252→T255
	M355 – S258	I359→V357→F462→S463→L471→H472→G473→V95→A96→D98→T100→P108→D109→A111→V128→D181→I204→A205→Y206→L252→T255
Mutants	I47 – S258	T48→L43→Q44→K45→L46→T48→N49→V50→N53→W153→A154→R213
	F289 – S258	F290→M295→R335→G339→H340→H381→F383→T384→G386→Q106→V107→G112→T113→S175→Y178→Y206→Q207→L208
	I355 – S258	I359→V375→T176→A460→L97→S98→T100→T113→A114→A176→A179→Q207→L208→M209

The comparison of allosteric pathways reveals several important insights. First, by using the same starting mutational sites and ending points, the mutant TNSALPs exhibit shorter allosteric pathways, which is in accordance with our hypothesis that the mutants caused by severe mutations have higher allosteric propensities. The system with the largest reduction in signaling path nodes is the mutant caused by M355I, which has the highest ΔBC and a relatively small ΔΔG. In WT, the path from M355 to S258 passed through the nodes F462→S463→L471→H472→G473 on the dimeric interface of the chain with mutations and the active sites P108 and D109 in the other chain (Fig 8A). In contrast, none of these sites participated in the signaling paths in the mutants. Hence the whole length becomes shorter and straight (Fig 8B). The same situation occurs in the path from M355I to N190 (Fig 8C and 8D). Second, the pathways in mutants involve fewer active sites, interfacial residues, and mutational sites. This comparison further highlights these functional regions as hubs for long-range allosteric communication in WTs, while it also means that the functional role of these reduced nodes may be lost if a severe mutation occurs. An extreme situation is observed for N47I, in which the path from I47 to S258 does not pass through the active area and employs the shortest and most straight path. Last, the path patterns are more robust in WT than in mutant states, suggesting that severe mutations introduce more pathway plasticity. For example, the shortest pathways from residues N47, L289, and M355 to N190 of the other chain in the WT shared some similar nodes, including P108, D109, G126, R184, D185, Y187, and S188, in the other chain, with most of located at the active site or ion binding pocket, and with all crossing the same area and to the ending residue N190 (orange circle region in Fig 8C and 8D). However, the situation was different in mutants. Fewer residues were shared in the three mutants, and only residue P108 showed the shortest pathway from I47, F289, and I355 to N190. The situation was similar in the paths from residues I47, F289, and I355 to S258 of the other chain. Three paths shared residues I204, A205, Y206, L252, and T255 of the other chain in the WT and none in the mutants.

**Fig 9:**
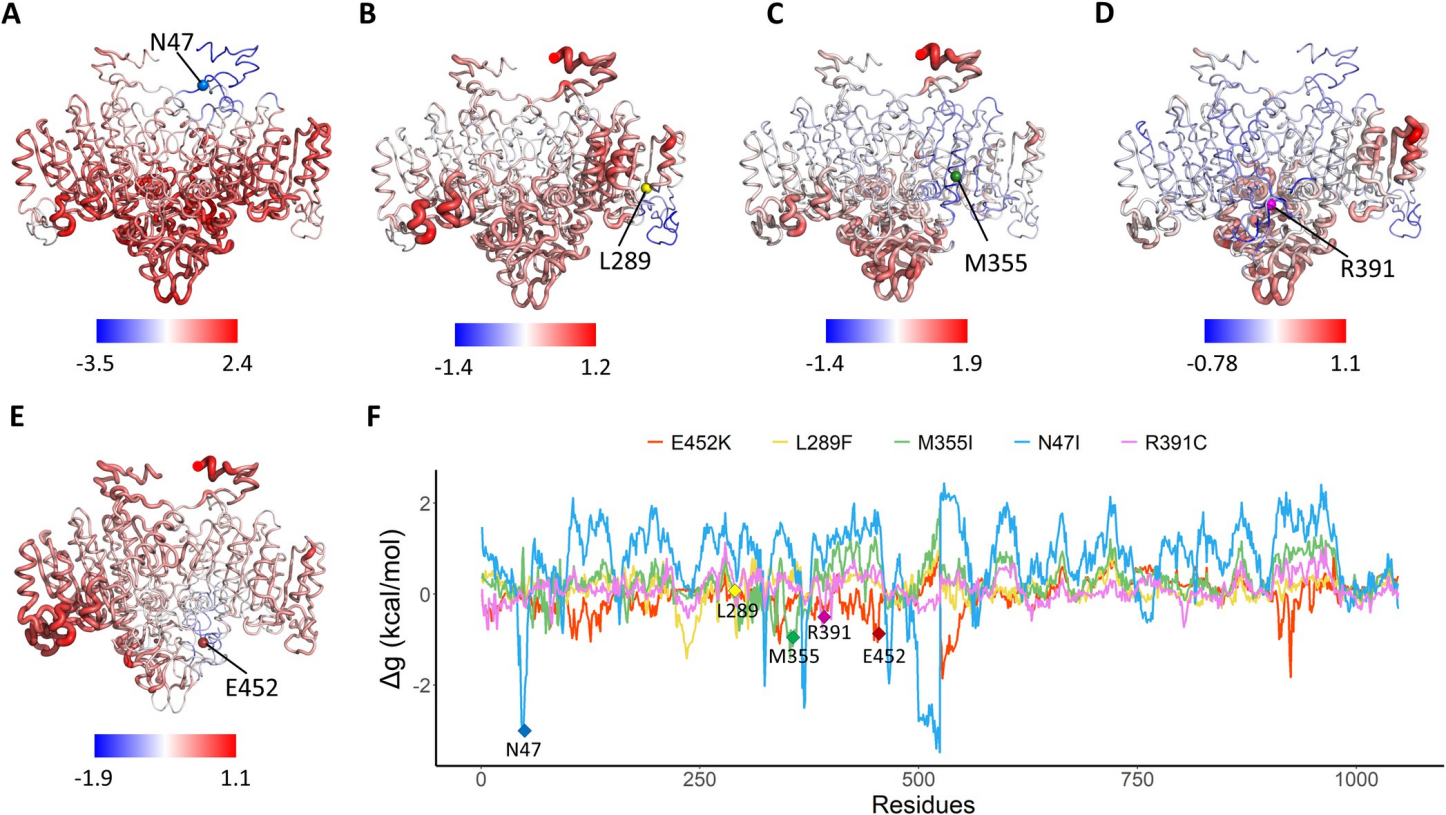
Allosteric effects of three studied severe mutations (N47I, L289F and M355I) and two predicted severe mutations (R391C and E452K) calculated by AlloSigMA. Cartoon structures of the TNSALP protein colored according to their free energy values obtained for the cases of (A) N47I, (B) L289F, (C) M355I, (D) R391C and (E) E452K, while blue color indicates negative allosteric free energy and red color indicates positive modulation. (F) Their free energy profiles are illustrated graphically with the residue index (chain *A*: 1–524; chain *B*: 525–1048) on the *x*-axis and Δg value on the *y*-axis. Blue, yellow, green, pink and red profiles represent the results for N47I, L289F, M355I, R391C and E452K, respectively.

As shown in Fig 8A, among the four mutations (E451K, M355I, R391C, and Y117C) with the largest ΔBC but low ΔΔG values, only M355I was related to the severe HPP phenotype. At the time of waiting, the ALPL mutation database has been updated by including several more severe mutations. We were surprised to find that the new patients with severe phenotypes related to E452K and R391C were reported to have mild and control mutations. To further investigate their allosteric effects and whether they are an intrinsic dynamic of these severe mutational sites, single-residue perturbation analysis was performed by using the AlloSigMA. The results showed that perturbations of all these five mutational sites induced intermolecular allosteric effects (Fig 9) in the form of both positive and negative modulation with maximum values of Δg (N47→chain *B*) = 2.44 kcal/mol, Δg (N289→chain *B*) = 1.07 kcal/mol, Δg (N335→chain *B*) = 0.99 kcal/mol, Δg (R391→chain *B*) = 1.05 kcal/mol and Δg (E452→chain *B*) = 0.86 kcal/mol, respectively. Although ΔΔG and Δg have different biological means referring to folding stability and allosteric ability, the comparison of them was also performed for these five mutations (S8 Fig). The most interesting finding is that N47I has smallest ΔΔG but large Δg, which is in agreement with MD simulation result that the mutant is stable but show most different allosteric landscapes. The analysis exhibited an evident influence on the stability of some functional regions, including the Ca^2+^ binding and crown domain, thereby revealing that severe mutations can induce changes in the stability of other sites and affect the catalytic activity of proteins.

Taken together, the MD results suggested that severe *ALPL* mutations may affect the signal transduction pathway between the two monomers of TNSALP. Allosteric pathways in WTs are robust and involve some functional sites consisting of active, interfacial, and mutational residues. In the allosteric mutant states, the overall pathway pattern is more “flexible”, with shorter pathways involving fewer functional sites, resulting in a severe phenotype. The allosteric free energy calculation once again suggest that ΔBC is a good and facile indictor for predicting severe mutations, whose molecular pathogenicity does not alter the local stability at active sites to change the protein folding energy but instead generates alternative and long-range molecular effects.

### Machine learning identifies key molecular features for classification of different mutation types

**Fig 10:**
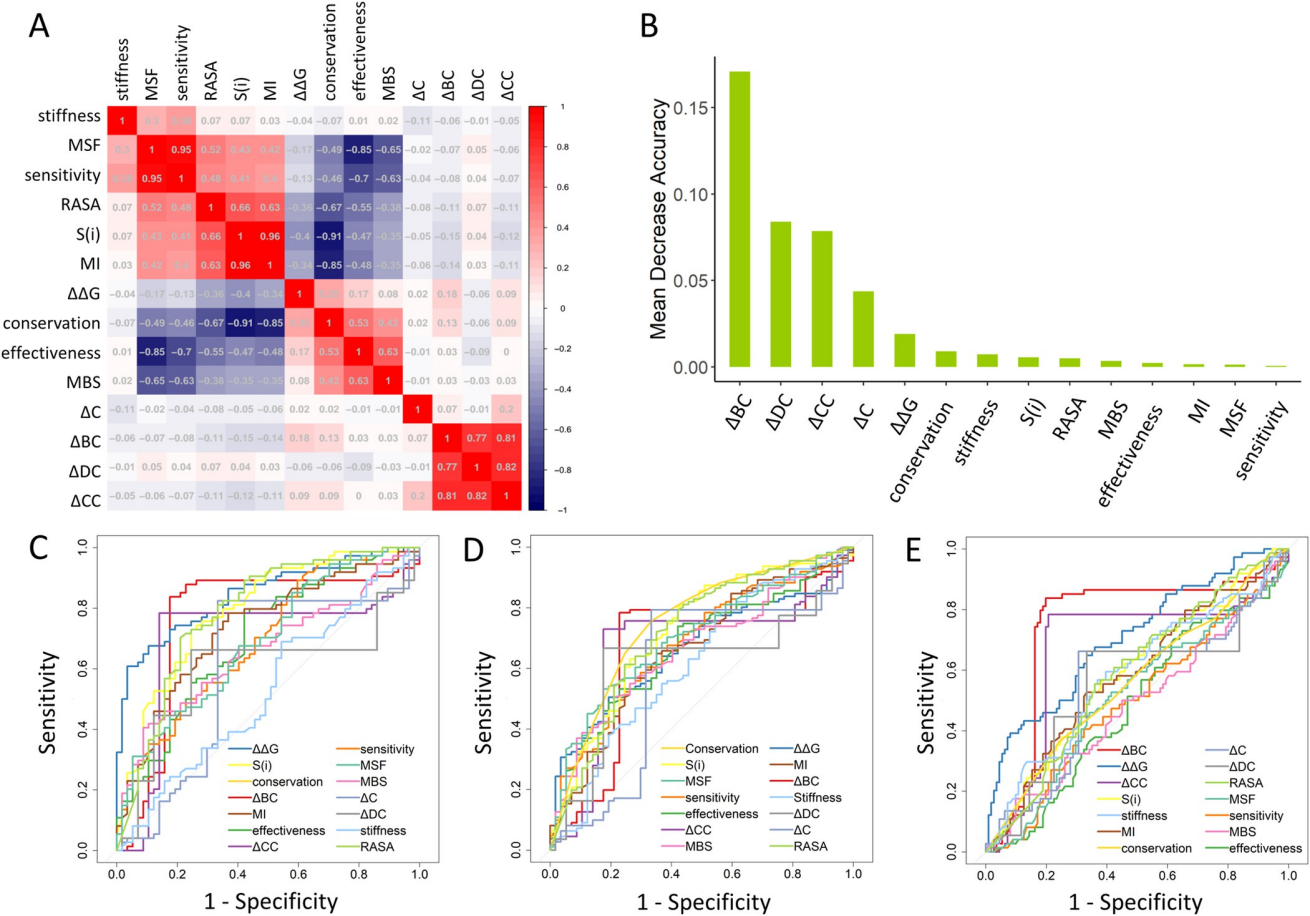
Performance evaluation of feature classification. (A) Heatmap of pairwise Spearman’s rank correlation coefficients between different features. (B) Feature importance of all used features ranked based on mean decrease accuracy in the RF classification. ROC curve AUCs for 14 features as a function of 1-specificity, including ROC curves for evaluating each feature in classifying *ALPL* mutations between (C) the control and severe groups, (D) control and mild groups, and (E) mild and severe groups.

To determine the relationship between the above studied features, their redundancy and ability to classify and predict various mutation types, we first computed the pairwise correlations between different prediction scores by using Spearman’s rank correlation coefficient (Fig 10A). These features can be classified into three major types according to their related sequence, network, and dynamics information. Among sequence-based features, ***S***(*i*) has high positive correlations with *MI* and RASA, but these three features all have negative correlations with conservation calculated by Consurf. For the four network parameters, ΔDC, ΔBC and ΔCC show significant correlations; only ΔC is an independent factor. The dynamics-based matrices that show more complex correlations have been divided into two groups: stiffness, MSF, and sensitivity; effectiveness and MBS. A highly positive correlation was predicted within each group, but a negative correlation was found between the two groups. The special index ΔΔG did not show any correlation with other features. Accordingly, these features could complement our understanding of the molecular signatures of HPP-causing mutations.

Next, we employed Radom Forest (RF) model to classify mild, severe, and control groups using the 14 features mentioned above (see S2 Text for details). We considered classifying the model containing five variables, whose accuracy of repeated cross-validation was 80.5%, as the optimized model, which was used for comparison with the model using all features. To be more specific, the feature importance was ranked by the mean decrease accuracy (Fig 10B). Most interestingly, we found that the top four important features were all network-based features, including ΔDC, ΔBC, ΔCC, and ΔC, suggesting an essential role of protein network topology in controlling mutation events. ΔΔG is an important feature and is also an important determinant of protein stability in mutations. To our surprise, features relating to ENM protein dynamics were not ranked as important predictors. This may be caused by the loopy structure of the TNSALP protein. By considering only the top five features, the RF model yielded an accuracy of 96.8%. However, taking all features into account, the accuracy of the RF model dropped to 92.2%. The inclusion of more features reduces the predictive accuracy of the RF model, which led us to focus on the specific interpretability of each parameter.

To aid in the interpretability of the classification effect of each parameter, we further evaluated the performance of the 14 features of the dataset composed of mild and severe mutations, mild and control mutations, and severe and control mutations. To this end, the ROC curves with the AUCs of the 14 metrics for each comparison group were plotted, and the related values are listed in Table 2. First, we examined the difference in the AUCs for the 14 functional features of the severe and control groups (Fig 10C); ΔΔG showed appreciable performance with an AUC of 0.8447. Moreover, the ***S***(*i*), conservation score, and ΔBC also showed good performance with AUCs, all greater than 0.73. We then tested the performance of the 14 features between mild and control group mutations (Fig 10D), as well as the mild and severe group mutations (Fig 10E). In the classification of the mild group and control group, two sequence-based and four dynamics-based parameters showed moderate predictive performance. The AUCs of the conservation score, entropy, MSF, effectiveness, sensitivity, and MBS were 0.7493, 0.7204, 0.7023, 0.6709, 0.6802, and 0.6666, respectively. In contrast, in the classification of the mild and severe groups, we found that ΔBC showed the best performance, with an AUC = 0.7471, while ΔΔG showed the second-best performance, with an AUC = 0.7048. The most interesting finding is that ΔΔG is a good indicator for identifying disease mutations, while ΔBC is the best indicator for classifying mild and severe mutations. The machine learning analysis showed that sequence, dynamics and network features contribute to classification of *ALPL* mutations and their phenotypes.

**Table 2:** The AUCs of 14 characteristics or parameters in each comparison group. The characteristics that performed best in each group are highlighted in red.

Features	Mild vs control	Severe vs control	Mild vs severe
conservation	0.7493	0.7927	0.5632
S(i)	0.7204	0.7934	0.5906
MI	0.6633	0.7336	0.5841
RASA	0.5284	0.5043	0.5329
ΔΔG	0.6641	0.8447	0.7048
ΔDC	0.6185	0.5991	0.5538
ΔBC	0.6618	0.7636	0.7471
ΔCC	0.6675	0.6961	0.6682
ΔC	0.5886	0.6081	0.5539
MSF	0.7023	0.6893	0.5140
effectiveness	0.6709	0.7009	0.4850
sensitivity	0.6802	0.6957	0.4968
MBS	0.6666	0.6632	0.4893
stiffness	0.619	0.5281	0.5860

## Discussion

As the biological hallmark of HPP, the reduced activity of TNSALP is caused by loss-of-function mutations in the *ALPL* gene; varying levels of reduced activity are related to mild or severe HPP phenotypes. Despite some progress, more research is needed to obtain a comprehensive understanding of genotype-phenotype interrelationships in HPP. Thus, in this study, the functional landscape of *ALPL* mutations was established by investigating a set of features generated from the protein sequence, network topology, and ENM dynamics calculation and their relationship with different HPP phenotypes. Further structure-function studies including MD simulation and structural-communication pathway analysis of mutant TNSALPs have supported our arguments that mutational hotspot sites often correspond to global mediators of allosteric interactions. In addition, a machine learning classifier was developed not only to examine the relationship between the pathogenicity status of mutations and their biophysical attributes, but also for the prediction of mutations in the control, mild, and severe groups. We have found that in addition to ΔΔG as the commonly used predictor, coevolutionary conservation and network-based features also yield strong signals in machine learning predictions and provide orthogonal information. These findings suggest that the study of molecular signatures and allosteric regulation of *ALPL* mutations may be a step toward defining a greater quantitative genotype-phenotype interrelationship in HPP.

Through the large-scale analysis of disease-causing *ALPL* mutations, we propose the following possible molecular principles underlying HPP-related mutations. First, HPP pathogenicity is largely due to the structural instability of TNSALP caused by *ALPL* mutations, which have variable effects on enzyme activity. Thus, for such cases, ΔΔG is a satisfactory and acceptable index for predicting pathogenic mutations, especially for distinguishing mutations between the control and severe groups. There is also the possibility of extrapolating our methods of using ΔΔG to estimate changes in protein stability upon mutations as a popular way to predict pathogenic mutations in other diseases. Many pervious works have proposed that BC and ΔBC are good network predictors for identifying important mutations [86,87]. Second, in our paper, we further demonstrate that ΔBC is also a good indicator to distinguish mild and severe groups in pathogenic mutations. We speculate that mutations in the severe group have stronger allosteric effects than mutations related to the milder forms of HPP, serving as “allosteric mutations” [88,89]. Analysis of allosteric properties of severe mutations adds an additional confirmatory layer to segregate variants in “mild” and “severe” states, thereby furthering understanding of the allosteric basis of loss-of-function mutations. Third, for the classification of mild mutations and differentiation from the control group, coevolutionary conservation has been shown to be the most important predictor, thereby suggesting that mild pathogenicity may be related to amino acid changes with small evolutionary substitution probability. However, to reach the elusive goal of establishing the precise relationship between *ALPL* mutation genotypes and HPP phenotypes and a more reliable prediction model or score, such as protein regulatory and functional binding site prediction [90], more clinical data on mutations [91] and data on enzyme activity [13] are needed.

## Supporting information

S1 Fig Distribution of ALPL mutations in terms of WT TNSALP sequence and structure. (A) The sequence overlaps between the control, mild and severe mutations. The WT sequence is at the bottom and the row for each type of amino acid. The mild, severe, and control phenotypes are colored with yellow, red and blue. (B) The mild, severe, and control mutation sites on the TNSALP WT structure are shown as spheres.
(TIF)

S2 Fig Evolutionary conservation analysis performed for TNSALP protein sequence 1to 524 aa using ConSurf. The amino acids are colored based on their conservation grades and conservation levels. A grade of 1 indicates rapidly evolving (variable) sites, which are color-coded in turquoise; 5 indicates sites that are evolving at an average rate, which are colored white; and 9 indicates slowly evolving (evolutionarily conserved) sites, which are color-coded in maroon. If the interval in a specific position spans 4 or more color grades the score is considered as unreliable. Such positions are colored light yellow in the graphic visualization output.
(TIF)

S3 Fig Comparisons of different molecular signatures for ALPL mutations. (A) The high correlation between entropy ***S***(*i*) and coevolution MI, and there are relative weak negative correlations (B) between ΔΔG and RASA, (C) between ΔΔG and ***S***(*i*), (D) between ΔΔG and *MI*.
(TIF)

S4 Fig 3D structure quality assessment of TNSALP modelled structures using Verify3D (B), PROHECK (C), ProSA (D and E) and ERRAT (F). Majority of residues within the N/C-terminals and loop regions exhibited high ERRAT values, low Verify 3D values and bad Psi degrees. Verify-3D analysis with 91.37% of the amino acids scoring > 0.2 in the 3D/1D profile; Only 5% of all the residues showed bad Psi degrees, and majority of which located in the terminal loops indicated that the model is well constructed. ProSA Z-score of − 9.07 shows the Z-value of the protein was similar to native protein of equivalent sizes(D). The reliability of the model was also shown by the ProSA energy plot with no obvious problematic regions with a positive value in the ProSA energy plot(E). Besides, the ERRAT plot is expressed as percentage of protein with calculated error value falls below the 95% rejection limit, and an ERRAT score of 84.97 indicates a good quality model.
(TIF)

S5 Fig Conformational dynamics of TNSALPs. (A-D) RMSDs for WT and mutated TNSALP (N47I, L289F and M355I) during the MD simulations, and (E-H) RMSF results for α-carbon atoms of four TNSALP systems. For each system, three independent replicas (1,2,3) of 500 ns were performed, and the results are shown in blue (replica 1^st^), red (replica 2^nd^) and green (replica 3^rd^), respectively.
(TIF)

S6 Fig DRN BC values of TNSALPs. DRN BC of WT and mutated TNSALP (N47I, L289F and M355I) during the three independent replicas of 500 ns MD simulations. For each system, the BC results are shown in blue (replica 1^st^), red (replica 2^nd^) and green (replica 3^rd^), respectively.
(TIF)

S7 Fig Comparison of BC values of AACEN and DRN. For each system, a replicas of 500 ns was singled out to compare the BC values of the two different networks of L289F (A) mutant and M355I (B) mutant. The green and grey line shown the BC values of residues of DRN and AACEN. If the residues corresponding to the peaks of DRN BC has the mild and severe mutations we collected, it is highlighted as yellow and red diamonds, respectively. DRNs can capture more peaks that correspond to disease mutations, such as T68M, T165I and I395V (mild mutations) and G334D (severe mutations) in L289F mutant, while T68M, V95M, T165I, D378V and L414M (mild mutations) and D378V (severe mutations) in M355I mutant.
(TIF)

S8 Fig Graphical comparison of ΔΔG (the green line) and Δg (the orange line) for the choose severe mutations.
(TIF)

S1 Table Collected mutation data of three kinds of phenotypes, with their structural domain annotation and various computational molecular signatures. (XLSX)

S1 Text Sequence and Structural Landscape of ALPL mutations. (DOCX)

S2 Text Machine Learning Models for Feature Selection. (DOCX)

## References

[1] Rathbun JC. Hypophosphatasia; a new developmental anomaly. American journal of diseases of children. 1948;75(6):822–831. doi: 10.1001/archpedi.1948.02030020840003 18110134

[2] Mornet E. Hypophosphatasia. Metabolism: clinical and experimental. 2018;82:142–155. doi: 10.1016/j.metabol.2017.08.013 28939177

[3] Komaru K, Ishida-Okumura Y, Numa-Kinjoh N, Hasegawa T, Oda K. Molecular and cellular basis of hypophosphatasia. Journal of oral biosciences. 2019;61(3):141–148. doi: 10.1016/j.job.2019.07.003 31400546

[4] Whyte MP, Zhang F, Wenkert D, McAlister WH, Mack KE, Benigno MC, et al. Hypophosphatasia: validation and expansion of the clinical nosology for children from 25 years experience with 173 pediatric patients. Bone. 2015;75:229–239. doi: 10.1016/j.bone.2015.02.022 25731960

[5] Vimalraj S. Alkaline phosphatase: Structure, expression and its function in bone mineralization. Gene. 2020;754:144855. doi: 10.1016/j.gene.2020.144855 32522695

[6] Millan JL, Whyte MP. Alkaline Phosphatase and Hypophosphatasia. Calcified tissue international. 2016;98(4):398–416. doi: 10.1007/s00223-015-0079-1 26590809 PMC4824800

[7] Whyte MP, Greenberg CR, Salman NJ, Bober MB, McAlister WH, Wenkert D, et al. Enzyme-replacement therapy in life-threatening hypophosphatasia. The New England journal of medicine. 2012;366(10):904–913. doi: 10.1056/NEJMoa1106173 22397652

[8] Bianchi ML, Vai S. Alkaline Phosphatase Replacement Therapy. Advances in experimental medicine and biology. 2019;1148:201–232. doi: 10.1007/978-981-13-7709-9_10 31482501

[9] Kyostila K, Syrja P, Lappalainen AK, Arumilli M, Hundi S, Karkamo V, et al. A homozygous missense variant in the alkaline phosphatase gene ALPL is associated with a severe form of canine hypophosphatasia. Sci Rep-Uk. 2019;9. doi: 10.1038/s41598-018-37801-2 30700765 PMC6353930

[10] Bianchi ML. Hypophosphatasia: an overview of the disease and its treatment. Osteoporosis international: a journal established as result of cooperation between the European Foundation for Osteoporosis and the National Osteoporosis Foundation of the USA. 2015;26(12):2743–2757. doi: 10.1007/s00198-015-3272-1 26245849

[11] Whyte MP. Hypophosphatasia—aetiology, nosology, pathogenesis, diagnosis and treatment. Nature reviews Endocrinology. 2016;12(4):233–246. doi: 10.1038/nrendo.2016.14 26893260

[12] Silvent J, Gasse B, Mornet E, Sire JY. Molecular evolution of the tissue-nonspecific alkaline phosphatase allows prediction and validation of missense mutations responsible for hypophosphatasia. The Journal of biological chemistry. 2014;289(35):24168–24179. doi: 10.1074/jbc.M114.576843 25023282 PMC4148848

[13] del Angel G, Reynders J, Negron C, Steinbrecher T, Mornet E. Large-scale in vitro functional testing and novel variant scoring via protein modeling provide insights into alkaline phosphatase activity in hypophosphatasia. Hum Mutat. 2020. doi: 10.1002/humu.24010 32160374 PMC7317754

[14] Mornet E, Taillandier A, Domingues C, Dufour A, Benaloun E, Lavaud N, et al. Hypophosphatasia: a genetic-based nosology and new insights in genotype-phenotype correlation. European journal of human genetics: EJHG. 2020. doi: 10.1038/s41431-020-00732-6 32973344 PMC7868366

[15] Zaher DM, El-Gamal MI, Omar HA, Aljareh SN, Al-Shamma SA, Ali AJ, et al. Recent advances with alkaline phosphatase isoenzymes and their inhibitors. Archiv der Pharmazie. 2020;353(5):e2000011. doi: 10.1002/ardp.202000011 32128876

[16] Mornet E, Stura E, Lia-Baldini AS, Stigbrand T, Menez A, Le Du MH. Structural evidence for a functional role of human tissue nonspecific alkaline phosphatase in bone mineralization. The Journal of biological chemistry. 2001;276(33):31171–31178. doi: 10.1074/jbc.M102788200 11395499

[17] Numa N, Ishida Y, Nasu M, Sohda M, Misumi Y, Noda T, et al. Molecular basis of perinatal hypophosphatasia with tissue-nonspecific alkaline phosphatase bearing a conservative replacement of valine by alanine at position 406. Structural importance of the crown domain. The FEBS journal. 2008;275(11):2727–2737. doi: 10.1111/j.1742-4658.2008.06414.x 18422967

[18] Hoylaerts MF, Manes T, Millan JL. Mammalian alkaline phosphatases are allosteric enzymes. The Journal of biological chemistry. 1997;272(36):22781–22787. doi: 10.1074/jbc.272.36.22781 9278439

[19] Martins L, de Almeida AB, Dos Santos EJL, Foster BL, Machado RA, Kantovitz KR, et al. A novel combination of biallelic ALPL mutations associated with adult hypophosphatasia: A phenotype-genotype association and computational analysis study. Bone. 2019;125:128–139. doi: 10.1016/j.bone.2019.05.005 31077853

[20] Borges B, Gallo G, Coelho C, Negri N, Maiello F, Hardy L, et al. Dynamic cross correlation analysis of Thermus thermophilus alkaline phosphatase and determinants of thermostability. Biochimica et biophysica acta General subjects. 2021;1865(7):129895. doi: 10.1016/j.bbagen.2021.129895 33781823

[21] Li Y, Song K, Zhang J, Lu SY. A Computational Method to Predict Effects of Residue Mutations on the Catalytic Efficiency of Hydrolases. Catalysts. 2021;11(2). doi: 10.3390/Catal11020286

[22] Tang N, Sandahl TD, Ott P, Kepp KP. Computing the Pathogenicity of Wilson’s Disease ATP7B Mutations: Implications for Disease Prevalence. J Chem Inf Model. 2019;59(12):5230–5243. doi: 10.1021/acs.jcim.9b00852 31751128

[23] Cheng N, Li M, Zhao L, Zhang B, Yang Y, Zheng CH, et al. Comparison and integration of computational methods for deleterious synonymous mutation prediction. Briefings in bioinformatics. 2020;21(3):970–981. doi: 10.1093/bib/bbz047 31157880

[24] Li G, Pahari S, Krishna Murthy A, Liang S, Fragoza R, Yu H, et al. SAAMBE-SEQ: A Sequence-based Method for Predicting Mutation Effect on Protein-protein Binding Affinity. Bioinformatics. 2020. doi: 10.1093/bioinformatics/btaa761 32866236 PMC8128451

[25] Kucukkal TG, Petukh M, Li L, Alexov E. Structural and physico-chemical effects of disease and non-disease nsSNPs on proteins. Current opinion in structural biology. 2015;32:18–24. doi: 10.1016/j.sbi.2015.01.003 25658850 PMC4511717

[26] Ghosh A, Vishveshwara S. A study of communication pathways in methionyl- tRNA synthetase by molecular dynamics simulations and structure network analysis. Proceedings of the National Academy of Sciences of the United States of America. 2007;104(40):15711–15716. doi: 10.1073/pnas.0704459104 17898174 PMC2000407

[27] Sirithep K, Xiao F, Raethong N, Zhang Y, Laoteng K, Hu G, et al. Probing Carbon Utilization of Cordyceps militaris by Sugar Transportome and Protein Structural Analysis. Cells. 2020;9(2). doi: 10.3390/cells9020401 32050592 PMC7072658

[28] Brinda KV, Vishveshwara S. A network representation of protein structures: implications for protein stability. Biophysical journal. 2005;89(6):4159–4170. doi: 10.1529/biophysj.105.064485 16150969 PMC1366981

[29] Achoch M, Dorantes-Gilardi R, Wymant C, Feverati G, Salamatian K, Vuillon L, et al. Protein structural robustness to mutations: an in silico investigation. Physical chemistry chemical physics: PCCP. 2016;18(20):13770–13780. doi: 10.1039/c5cp06091e 26688116

[30] Giollo M, Martin AJ, Walsh I, Ferrari C, Tosatto SC. NeEMO: a method using residue interaction networks to improve prediction of protein stability upon mutation. BMC genomics. 2014;15 Suppl 4:S7. doi: 10.1186/1471-2164-15-S4-S7 25057121 PMC4083412

[31] Amamuddy OS, Boateng RA, Barozi V, Nyamai DW, Bishop OT. Novel dynamic residue network analysis approaches to study allosteric modulation: SARS-CoV-2 M-pro and its evolutionary mutations as a case study. Comput Struct Biotec. 2021;19:6431–6455. doi: 10.1016/j.csbj.2021.11.016 34849191 PMC8613987

[32] Okeke CJ, Musyoka TM, Amamuddy OS, Barozi V, Bishop OT. Allosteric pockets and dynamic residue network hubs of falcipain 2 in mutations including those linked to artemisinin resistance. Comput Struct Biotec. 2021;19:5647–5666. doi: 10.1016/j.csbj.2021.10.011 34745456 PMC8545671

[33] Kumar A, Glembo TJ, Ozkan SB. The Role of Conformational Dynamics and Allostery in the Disease Development of Human Ferritin. Biophysical journal. 2015;109(6):1273–1281. doi: 10.1016/j.bpj.2015.06.060 26255589 PMC4576160

[34] Sayilgan JF, Haliloglu T, Gonen M. Protein dynamics analysis reveals that missense mutations in cancer-related genes appear frequently on hinge-neighboring residues. Proteins. 2019;87(6):512–519. doi: 10.1002/prot.25673 30785643

[35] Atilgan AR, Durell SR, Jernigan RL, Demirel MC, Keskin O, Bahar I. Anisotropy of fluctuation dynamics of proteins with an elastic network model. Biophysical journal. 2001;80(1):505–515. doi: 10.1016/S0006-3495(01)76033-X 11159421 PMC1301252

[36] Bahar I, Atilgan AR, Erman B. Direct evaluation of thermal fluctuations in proteins using a single-parameter harmonic potential. Folding & design. 1997;2(3):173–181. doi: 10.1016/S1359-0278(97)00024-2 9218955

[37] Frappier V, Najmanovich RJ. A coarse-grained elastic network atom contact model and its use in the simulation of protein dynamics and the prediction of the effect of mutations. PLoS computational biology. 2014;10(4):e1003569. doi: 10.1371/journal.pcbi.1003569 24762569 PMC3998880

[38] Ponzoni L, Bahar I. Structural dynamics is a determinant of the functional significance of missense variants. Proceedings of the National Academy of Sciences of the United States of America. 2018;115(16):4164–4169. doi: 10.1073/pnas.1715896115 29610305 PMC5910821

[39] Agajanian S, Odeyemi O, Bischoff N, Ratra S, Verkhivker GM. Machine Learning Classification and Structure-Functional Analysis of Cancer Mutations Reveal Unique Dynamic and Network Signatures of Driver Sites in Oncogenes and Tumor Suppressor Genes. J Chem Inf Model. 2018;58(10):2131–2150. doi: 10.1021/acs.jcim.8b00414 30253099

[40] Agajanian S, Oluyemi O, Verkhivker GM. Integration of Random Forest Classifiers and Deep Convolutional Neural Networks for Classification and Biomolecular Modeling of Cancer Driver Mutations. Frontiers in molecular biosciences. 2019;6:44. doi: 10.3389/fmolb.2019.00044 31245384 PMC6579812

[41] Verkhivker GM, Agajanian S, Hu G, Tao P. Allosteric Regulation at the Crossroads of New Technologies: Multiscale Modeling, Networks, and Machine Learning. Frontiers in molecular biosciences. 2020;7:136. doi: 10.3389/fmolb.2020.00136 32733918 PMC7363947

[42] Verkhivker GM. Biophysical simulations and structure-based modeling of residue interaction networks in the tumor suppressor proteins reveal functional role of cancer mutation hotspots in molecular communication. Biochimica et biophysica acta General subjects. 2019;1863(1):210–225. doi: 10.1016/j.bbagen.2018.10.009 30339916

[43] Stetz G, Astl L, Verkhivker GM. Exploring Mechanisms of Communication Switching in the Hsp90-Cdc37 Regulatory Complexes with Client Kinases through Allosteric Coupling of Phosphorylation Sites: Perturbation-Based Modeling and Hierarchical Community Analysis of Residue Interaction Networks. Journal of chemical theory and computation. 2020;16(7):4706–4725. doi: 10.1021/acs.jctc.0c00280 32492340

[44] Verkhivker G, Agajanian S, Oztas D, Gupta G. Dynamic Profiling of Binding and Allosteric Propensities of the SARS-CoV-2 Spike Protein with Different Classes of Antibodies: Mutational and Perturbation-Based Scanning Reveals the Allosteric Duality of Functionally Adaptable Hotspots. Journal of chemical theory and computation. 2021;17(7):4578–4598. doi: 10.1021/acs.jctc.1c00372 34138559

[45] Smith IN, Thacker S, Seyfi M, Cheng F, Eng C. Conformational Dynamics and Allosteric Regulation Landscapes of Germline PTEN Mutations Associated with Autism Compared to Those Associated with Cancer. American journal of human genetics. 2019;104(5):861–878. doi: 10.1016/j.ajhg.2019.03.009 31006514 PMC6506791

[46] Portelli S, Barr L, de Sa AGC, Pires DEV, Ascher DB. Distinguishing between PTEN clinical phenotypes through mutation analysis. Comput Struct Biotec. 2021;19:3097–3109. doi: 10.1016/j.csbj.2021.05.028 34141133 PMC8180946

[47] Murthy ASN, Suresh RV, Nallur BR. Comprehensive in silico mutational-sensitivity analysis of PTEN establishes signature regions implicated in pathogenesis of Autism Spectrum Disorders. Genomics. 2021;113(1):999–1017. doi: 10.1016/j.ygeno.2020.10.035 33152507

[48] Liang ZJ, Verkhivker GM, Hu G. Integration of network models and evolutionary analysis into high-throughput modeling of protein dynamics and allosteric regulation: theory, tools and applications. Briefings in bioinformatics. 2020;21(3):815–835. doi: 10.1093/bib/bbz029 30911759

[49] Astl L, Verkhivker GM. Data-driven computational analysis of allosteric proteins by exploring protein dynamics, residue coevolution and residue interaction networks. Biochimica et biophysica acta General subjects. 2019. doi: 10.1016/j.bbagen.2019.07.008 31330173

[50] Liang Z, Hu J, Yan W, Jiang H, Hu G, Luo C. Deciphering the role of dimer interface in intrinsic dynamics and allosteric pathways underlying the functional transformation of DNMT3A. Biochimica et biophysica acta General subjects. 2018;1862(7):1667–1679. doi: 10.1016/j.bbagen.2018.04.015 29674125

[51] Xiao F, Song X, Tian P, Gan M, Verkhivker GM, Hu G. Comparative Dynamics and Functional Mechanisms of the CYP17A1 Tunnels Regulated by Ligand Binding. J Chem Inf Model. 2020;60(7):3632–3647. doi: 10.1021/acs.jcim.0c00447 32530640

[52] Iqbal S, Perez-Palma E, Jespersen JB, May P, Hoksza D, Heyne HO, et al. Comprehensive characterization of amino acid positions in protein structures reveals molecular effect of missense variants. Proceedings of the National Academy of Sciences of the United States of America. 2020;117(45):28201–28211. doi: 10.1073/pnas.2002660117 33106425 PMC7668189

[53] Tack DS, Tonner PD, Pressman A, Olson ND, Levy SF, Romantseva EF, et al. The genotype-phenotype landscape of an allosteric protein. Molecular systems biology. 2021;17(3):e10179. doi: 10.15252/msb.202010179 33784029 PMC8009258

[54] Ashkenazy H, Abadi S, Martz E, Chay O, Mayrose I, Pupko T, et al. ConSurf 2016: an improved methodology to estimate and visualize evolutionary conservation in macromolecules. Nucleic Acids Res. 2016;44(W1):W344–350. doi: 10.1093/nar/gkw408 27166375 PMC4987940

[55] Mao W, Kaya C, Dutta A, Horovitz A, Bahar I. Comparative study of the effectiveness and limitations of current methods for detecting sequence coevolution. Bioinformatics. 2015;31(12):1929–1937. doi: 10.1093/bioinformatics/btv103 25697822 PMC4481699

[56] Bakan A, Dutta A, Mao W, Liu Y, Chennubhotla C, Lezon TR, et al. Evol and ProDy for bridging protein sequence evolution and structural dynamics. Bioinformatics. 2014;30(18):2681–2683. doi: 10.1093/bioinformatics/btu336 24849577 PMC4155247

[57] Marti-Renom MA, Stuart AC, Fiser A, Sanchez R, Melo F, Sali A. Comparative protein structure modeling of genes and genomes. Annual review of biophysics and biomolecular structure. 2000;29:291–325. doi: 10.1146/annurev.biophys.29.1.291 10940251

[58] Llinas P, Stura EA, Menez A, Kiss Z, Stigbrand T, Millan JL, et al. Structural studies of human placental alkaline phosphatase in complex with functional ligands. Journal of molecular biology. 2005;350(3):441–451. doi: 10.1016/j.jmb.2005.04.068 15946677

[59] Eisenberg D, Luthy R, Bowie JU. VERIFY3D: Assessment of protein models with three-dimensional profiles. In: Carter CW, Sweet RM, editors. MACROMOLECULAR CRYSTALLOGRAPHY, PT B. 2771997. p. 396–404. doi: 10.1016/s0076-6879(97)77022-8 9379925

[60] Laskowski RA, Macarthur MW, Moss DS, Thornton JM. PROCHECK—A PROGRAM TO CHECK THE STEREOCHEMICAL QUALITY OF PROTEIN STRUCTURES. JOURNAL OF APPLIED CRYSTALLOGRAPHY. 1993;26:283–291. doi: 10.1107/S0021889892009944

[61] Wiederstein M, Sippl MJ. ProSA-web: interactive web service for the recognition of errors in three-dimensional structures of proteins. Nucleic acids research. 2007;35:W407–W410. doi: 10.1093/nar/gkm290 17517781 PMC1933241

[62] Colovos C, Yeates TO. VERIFICATION OF PROTEIN STRUCTURES—PATTERNS OF NONBONDED ATOMIC INTERACTIONS. PROTEIN SCIENCE. 1993;2(9):1511–1519. doi: 10.1002/pro.5560020916 8401235 PMC2142462

[63] Zhang Z, Wang L, Gao Y, Zhang J, Zhenirovskyy M, Alexov E. Predicting folding free energy changes upon single point mutations. Bioinformatics. 2012;28(5):664–671. doi: 10.1093/bioinformatics/bts005 22238268 PMC3289912

[64] Besterman AD, Althoff T, Elfferich P, Gutierrez-Mejia I, Sadik J, Bernstein JA, et al. Functional and structural analyses of novel Smith-Kingsmore Syndrome-Associated MTOR variants reveal potential new mechanisms and predictors of pathogenicity. PLoS genetics. 2021;17(7):e1009651. doi: 10.1371/journal.pgen.1009651 34197453 PMC8279410

[65] Mihel J, Sikic M, Tomic S, Jeren B, Vlahovicek K. PSAIA—Protein structure and interaction analyzer. Bmc Struct Biol. 2008;8. doi: 10.1186/1472-6807-8-21 18400099 PMC2364630

[66] Yan W, Hu G, Liang Z, Zhou J, Yang Y, Chen J, et al. Node-Weighted Amino Acid Network Strategy for Characterization and Identification of Protein Functional Residues. J Chem Inf Model. 2018;58(9):2024–2032. doi: 10.1021/acs.jcim.8b00146 30107728

[67] Yan W, Sun M, Hu G, Zhou J, Zhang W, Chen J, et al. Amino acid contact energy networks impact protein structure and evolution. Journal of theoretical biology. 2014;355:95–104. doi: 10.1016/j.jtbi.2014.03.032 24703984

[68] Zhang C, Kim SH. Environment-dependent residue contact energies for proteins. Proceedings of the National Academy of Sciences of the United States of America. 2000;97(6):2550–2555. doi: 10.1073/pnas.040573597 10706611 PMC15966

[69] Doncheva NT, Klein K, Domingues FS, Albrecht M. Analyzing and visualizing residue networks of protein structures. Trends in biochemical sciences. 2011;36(4):179–182. doi: 10.1016/j.tibs.2011.01.002 21345680

[70] Bakan A, Meireles LM, Bahar I. ProDy: protein dynamics inferred from theory and experiments. Bioinformatics. 2011;27(11):1575–1582. doi: 10.1093/bioinformatics/btr168 21471012 PMC3102222

[71] Atilgan C, Atilgan AR. Perturbation-response scanning reveals ligand entry-exit mechanisms of ferric binding protein. PLoS computational biology. 2009;5(10):e1000544. doi: 10.1371/journal.pcbi.1000544 19851447 PMC2758672

[72] Tyner S, Briatte F, Hofmann H. Network Visualization with ggplot2. R J. 2017;9(1):27–59.

[73] Bussi G, Donadio D, Parrinello M. Canonical sampling through velocity rescaling. J Chem Phys. 2007;126(1). doi: 10.1063/1.2408420 17212484

[74] Van Der Spoel D, Lindahl E, Hess B, Groenhof G, Mark AE, Berendsen HJ. GROMACS: fast, flexible, and free. Journal of computational chemistry. 2005;26(16):1701–1718. doi: 10.1002/jcc.20291 16211538

[75] Lindorff-Larsen K, Piana S, Palmo K, Maragakis P, Klepeis JL, Dror RO, et al. Improved side-chain torsion potentials for the Amber ff99SB protein force field. Proteins. 2010;78(8):1950–1958. doi: 10.1002/prot.22711 20408171 PMC2970904

[76] Essmann U, Perera L, Berkowitz ML, Darden T, Lee H, Pedersen LG. A Smooth Particle Mesh Ewald Method. J Chem Phys. 1995;103(19):8577–8593. doi: 10.1063/1.470117

[77] Hess B, Bekker H, Berendsen HJC, Fraaije JGEM. LINCS: A linear constraint solver for molecular simulations. Journal of computational chemistry. 1997;18(12):1463–1472. doi: 10.1002/(Sici)1096-987x(199709)18:12&lt;1463::Aid-Jcc4&gt;3.3.Co;2-L

[78] Brown DK, Penkler DL, Amamuddy OS, Ross C, Atilgan AR, Atilgan C, et al. MD-TASK: a software suite for analyzing molecular dynamics trajectories. Bioinformatics. 2017;33(17):2768–2771. doi: 10.1093/bioinformatics/btx349 28575169 PMC5860072

[79] Floyd RW. Algorithm-97—Shortest Path. Commun Acm. 1962;5(6):345–345. doi: 10.1145/367766.368168

[80] Guarnera E, Tan ZW, Zheng Z, Berezovsky IN. AlloSigMA: allosteric signaling and mutation analysis server. Bioinformatics. 2017;33(24):3996–3998. doi: 10.1093/bioinformatics/btx430 29106449

[81] Tan ZW, Guarnera E, Tee WV, Berezovsky IN. AlloSigMA 2: paving the way to designing allosteric effectors and to exploring allosteric effects of mutations. Nucleic Acids Res. 2020;48(W1):W116–W124. doi: 10.1093/nar/gkaa338 32392302 PMC7319554

[82] Hu G, Yan WY, Zhou JH, Shen BR. Residue interaction network analysis of Dronpa and a DNA clamp. Journal of theoretical biology. 2014;348:55–64. doi: 10.1016/j.jtbi.2014.01.023 24486230

[83] Sayilgan JF, Haliloglu T, Gonen M. Protein dynamics analysis identifies candidate cancer driver genes and mutations in TCGA data. Proteins. 2021. doi: 10.1002/prot.26054 33550612

[84] Kapetis D, Sassone J, Yang Y, Galbardi B, Xenakis MN, Westra RL, et al. Network topology of NaV1.7 mutations in sodium channel-related painful disorders. BMC systems biology. 2017;11(1):28. doi: 10.1186/s12918-016-0382-0 28235406 PMC5324268

[85] Leander M, Yuan Y, Meger A, Cui Q, Raman S. Functional plasticity and evolutionary adaptation of allosteric regulation. Proceedings of the National Academy of Sciences of the United States of America. 2020;117(41):25445–25454. doi: 10.1073/pnas.2002613117 32999067 PMC7568325

[86] Sanyanga TA, Nizami B, Tastan Bishop O. Mechanism of Action of Non-Synonymous Single Nucleotide Variations Associated with alpha-Carbonic Anhydrase II Deficiency. MOLECULES. 2019;24(21). doi: 10.3390/molecules24213987 31690045 PMC6864701

[87] Amusengeri A, Tata RB, Bishop OT. Understanding the Pyrimethamine Drug Resistance Mechanism via Combined Molecular Dynamics and Dynamic Residue Network Analysis. MOLECULES. 2020;25(4). doi: 10.3390/molecules25040904 32085470 PMC7070769

[88] Lu S, Qiu Y, Ni D, He X, Pu J, Zhang J. Emergence of allosteric drug-resistance mutations: new challenges for allosteric drug discovery. Drug discovery today. 2020;25(1):177–184. doi: 10.1016/j.drudis.2019.10.006 31634592

[89] Tan ZW, Tee WV, Guarnera E, Booth L, Berezovsky IN. AlloMAPS: allosteric mutation analysis and polymorphism of signaling database. Nucleic Acids Research. 2019;47(D1):D265–D270. doi: 10.1093/nar/gky1028 30365033 PMC6323965

[90] Mishra SK, Kandoi G, Jernigan RL. Coupling dynamics and evolutionary information with structure to identify protein regulatory and functional binding sites. Proteins. 2019;87(10):850–868. doi: 10.1002/prot.25749 31141211 PMC6718341

[91] Farris J, Calhoun B, Alam MS, Lee S, Haldar K. Large scale analyses of genotype-phenotype relationships of glycine decarboxylase mutations and neurological disease severity. PLoS computational biology. 2020;16(5):e1007871. doi: 10.1371/journal.pcbi.1007871 32421718 PMC7259800

