# Supplementary figures and images for: Dissecting mutational allosteric effects in alkaline phosphatases associated with different Hypophosphatasia phenotypes: An integrative computational investigation
Source: PLoS Comput Biol. 2022 Mar 23;18(3):e1010009. doi: 10.1371/journal.pcbi.1010009 (PMC8979438; doi:10.1371/journal.pcbi.1010009)

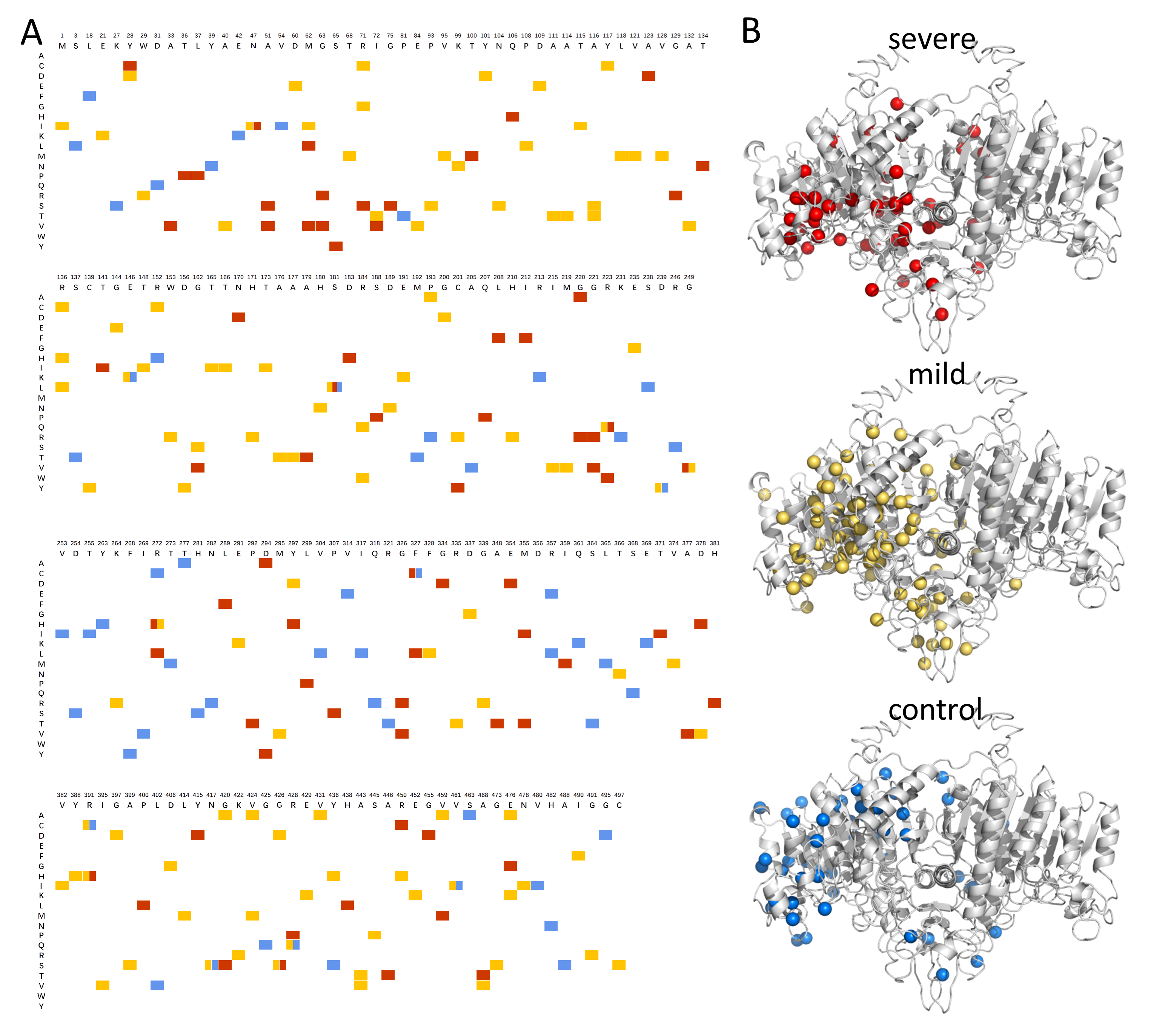

Supplement: S1 Fig — (A) The sequence overlaps between the control, mild and severe mutations. The WT sequence is at the bottom and the row for each type of amino acid. The mild, severe, and control phenotypes are colored with yellow, red and blue. (B) The mild, severe, and control mutation sites on the TNSALP WT structure are shown as spheres. (TIF) [file pcbi.1010009.s001.tif]

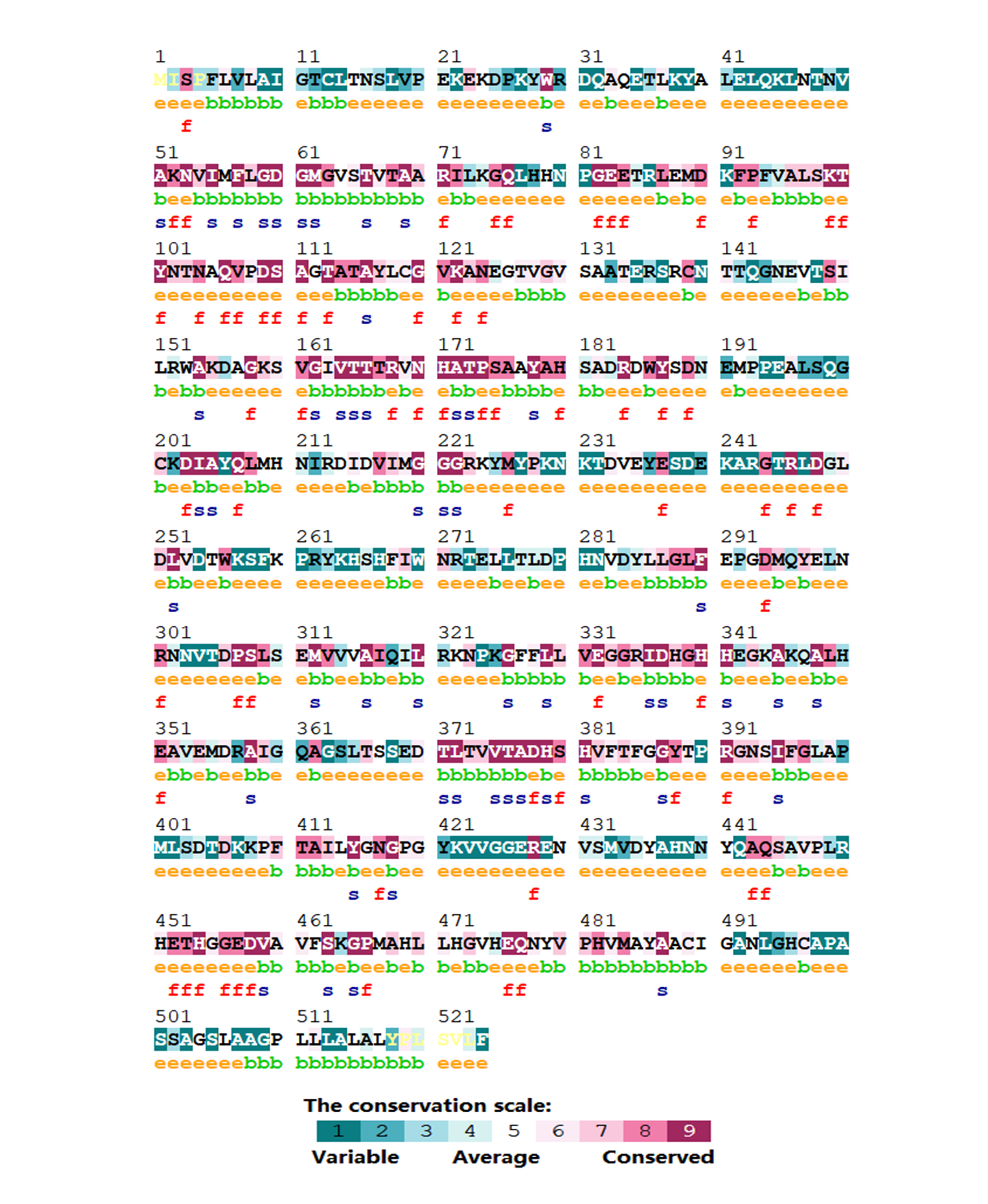

Supplement: S2 Fig — The amino acids are colored based on their conservation grades and conservation levels. A grade of 1 indicates rapidly evolving (variable) sites, which are color-coded in turquoise; 5 indicates sites that are evolving at an average rate, which are colored white; and 9 indicates slowly evolving (evolutionarily conserved) sites, which are color-coded in maroon. If the interval in a specific position spans 4 or more color grades the score is considered as unreliable. Such positions are colored light yellow in the graphic visualization output. (TIF) [file pcbi.1010009.s002.tif]

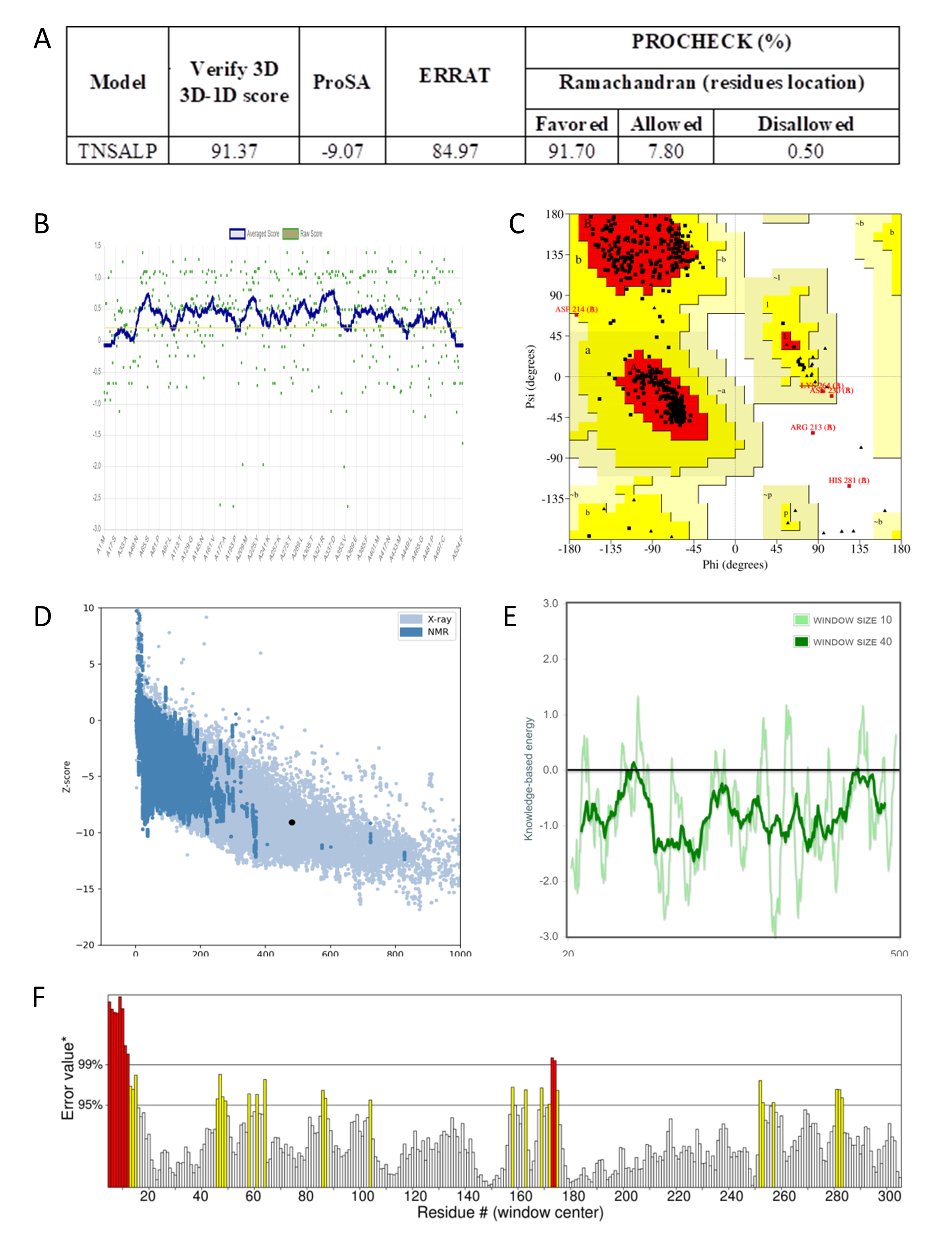

Supplement: S3 Fig — (A) The high correlation between entropy S(i) and coevolution MI, and there are relative weak negative correlations (B) between ΔΔG and RASA, (C) between ΔΔG and S(i), (D) between ΔΔG and MI. (TIF) [file pcbi.1010009.s003.tif]

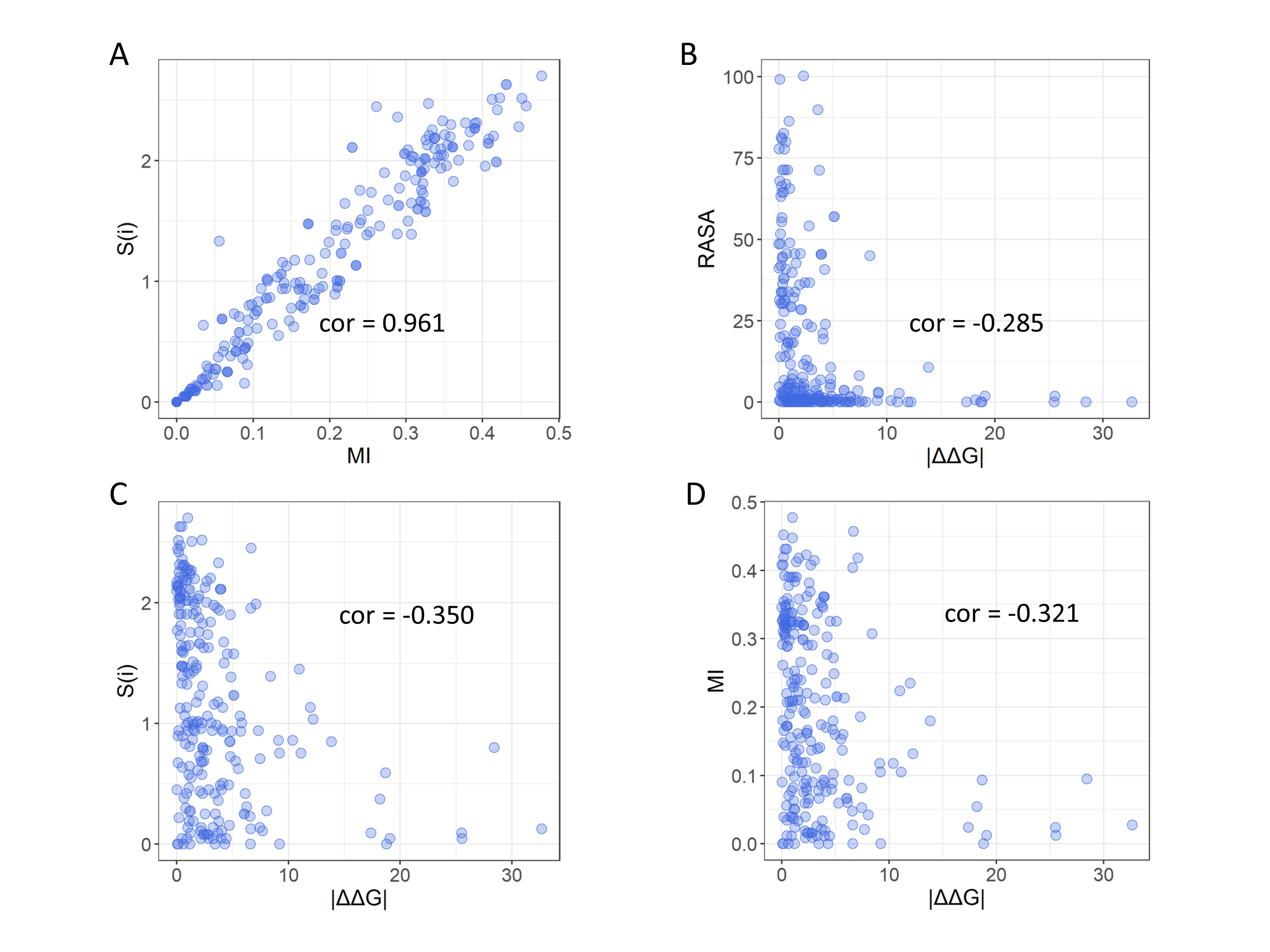

Supplement: S4 Fig — 3D structure quality assessment of TNSALP modelled structures using Verify3D (B), PROHECK (C), ProSA (D and E) and ERRAT (F). Majority of residues within the N/C-terminals and loop regions exhibited high ERRAT values, low Verify 3D values and bad Psi degrees. Verify-3D analysis with 91.37% of the amino acids scoring > 0.2 in the 3D/1D profile; Only 5% of all the residues showed bad Psi degrees, and majority of which located in the terminal loops indicated that the model is well constructed. ProSA Z-score of − 9.07 shows the Z-value of the protein was similar to native protein of equivalent sizes(D). The reliability of the model was also shown by the ProSA energy plot with no obvious problematic regions with a positive value in the ProSA energy plot(E). Besides, the ERRAT plot is expressed as percentage of protein with calculated error value falls below the 95% rejection limit, and an ERRAT score of 84.97 indicates a good quality model. (TIF) [file pcbi.1010009.s004.tif]

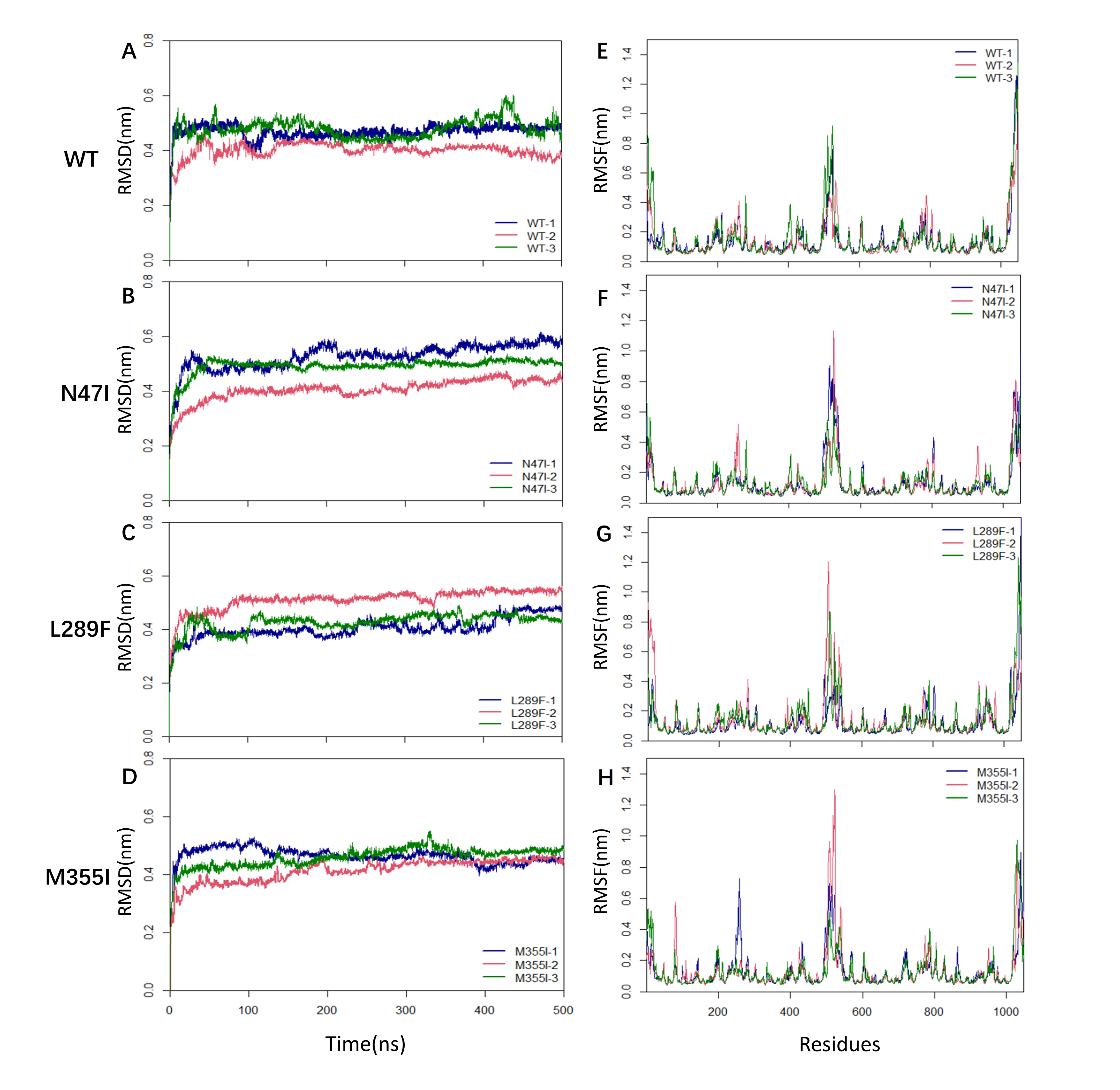

Supplement: S5 Fig — (A-D) RMSDs for WT and mutated TNSALP (N47I, L289F and M355I) during the MD simulations, and (E-H) RMSF results for α-carbon atoms of four TNSALP systems. For each system, three independent replicas (1,2,3) of 500 ns were performed, and the results are shown in blue (replica 1st), red (replica 2nd) and green (replica 3rd), respectively. (TIF) [file pcbi.1010009.s005.tif]

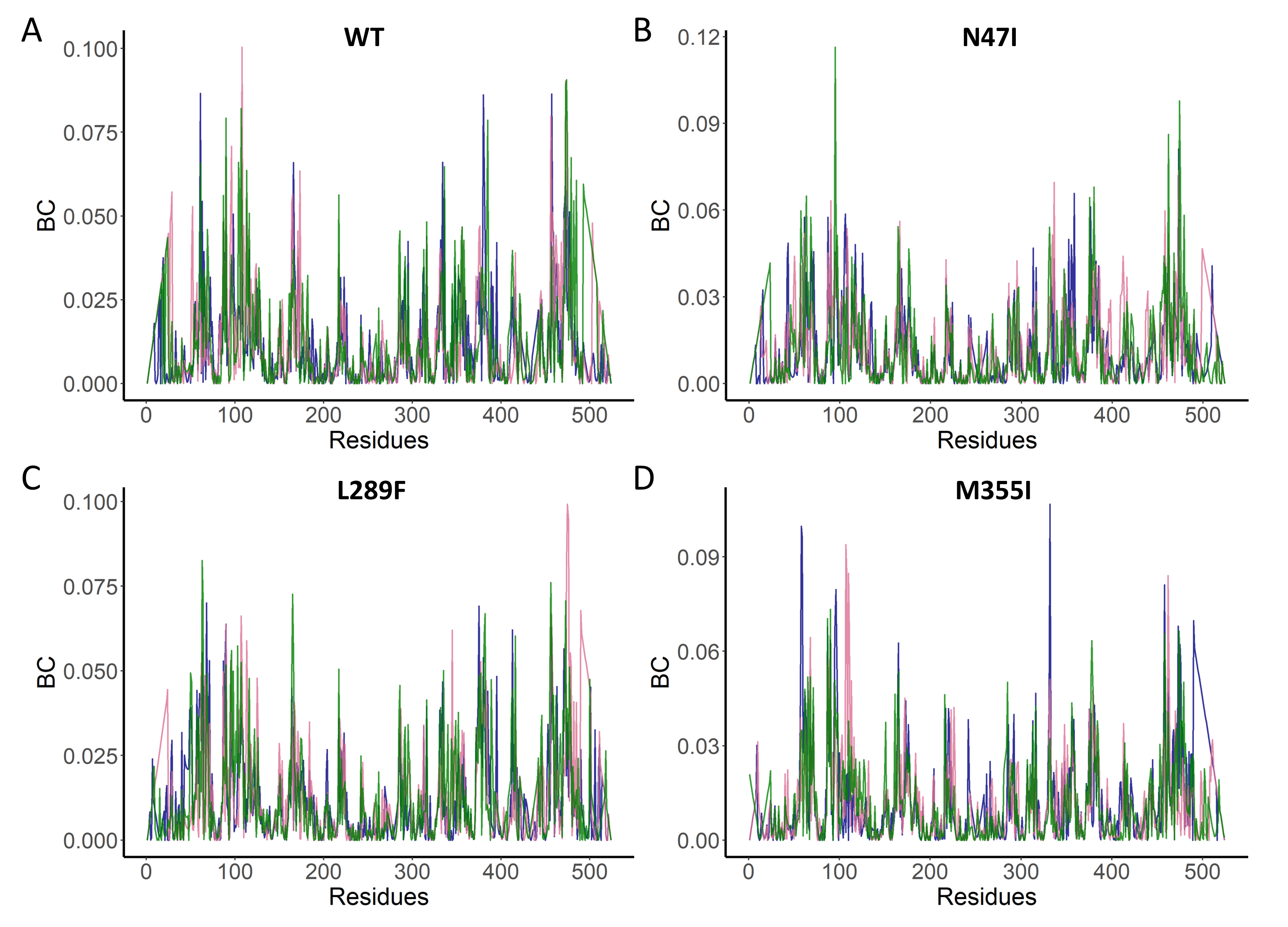

Supplement: S6 Fig — DRN BC of WT and mutated TNSALP (N47I, L289F and M355I) during the three independent replicas of 500 ns MD simulations. For each system, the BC results are shown in blue (replica 1st), red (replica 2nd) and green (replica 3rd), respectively. (TIF) [file pcbi.1010009.s006.tif]

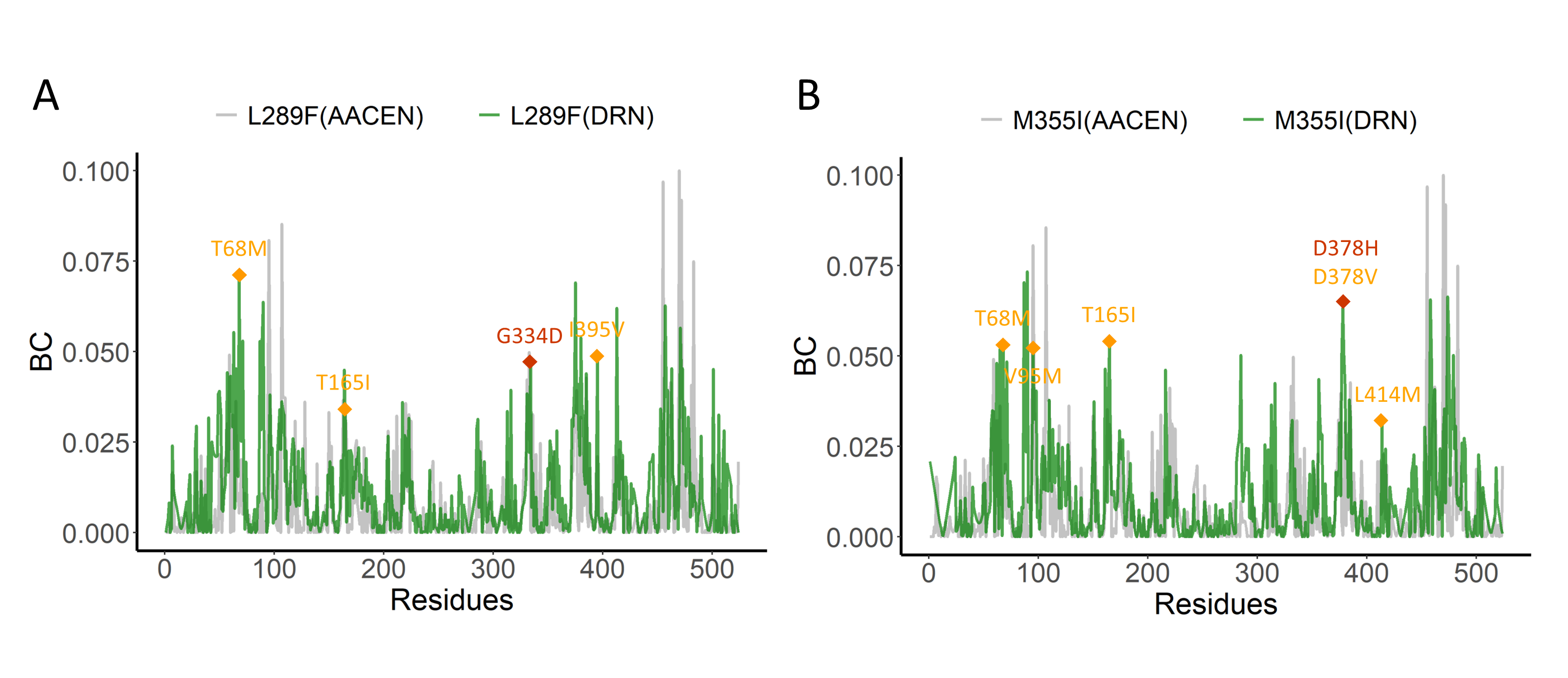

Supplement: S7 Fig — For each system, a replicas of 500 ns was singled out to compare the BC values of the two different networks of L289F (A) mutant and M355I (B) mutant. The green and grey line shown the BC values of residues of DRN and AACEN. If the residues corresponding to the peaks of DRN BC has the mild and severe mutations we collected, it is highlighted as yellow and red diamonds, respectively. DRNs can capture more peaks that correspond to disease mutations, such as T68M, T165I and I395V (mild mutations) and G334D (severe mutations) in L289F mutant, while T68M, V95M, T165I, D378V and L414M (mild mutations) and D378V (severe mutations) in M355I mutant. (TIF) [file pcbi.1010009.s007.tif]

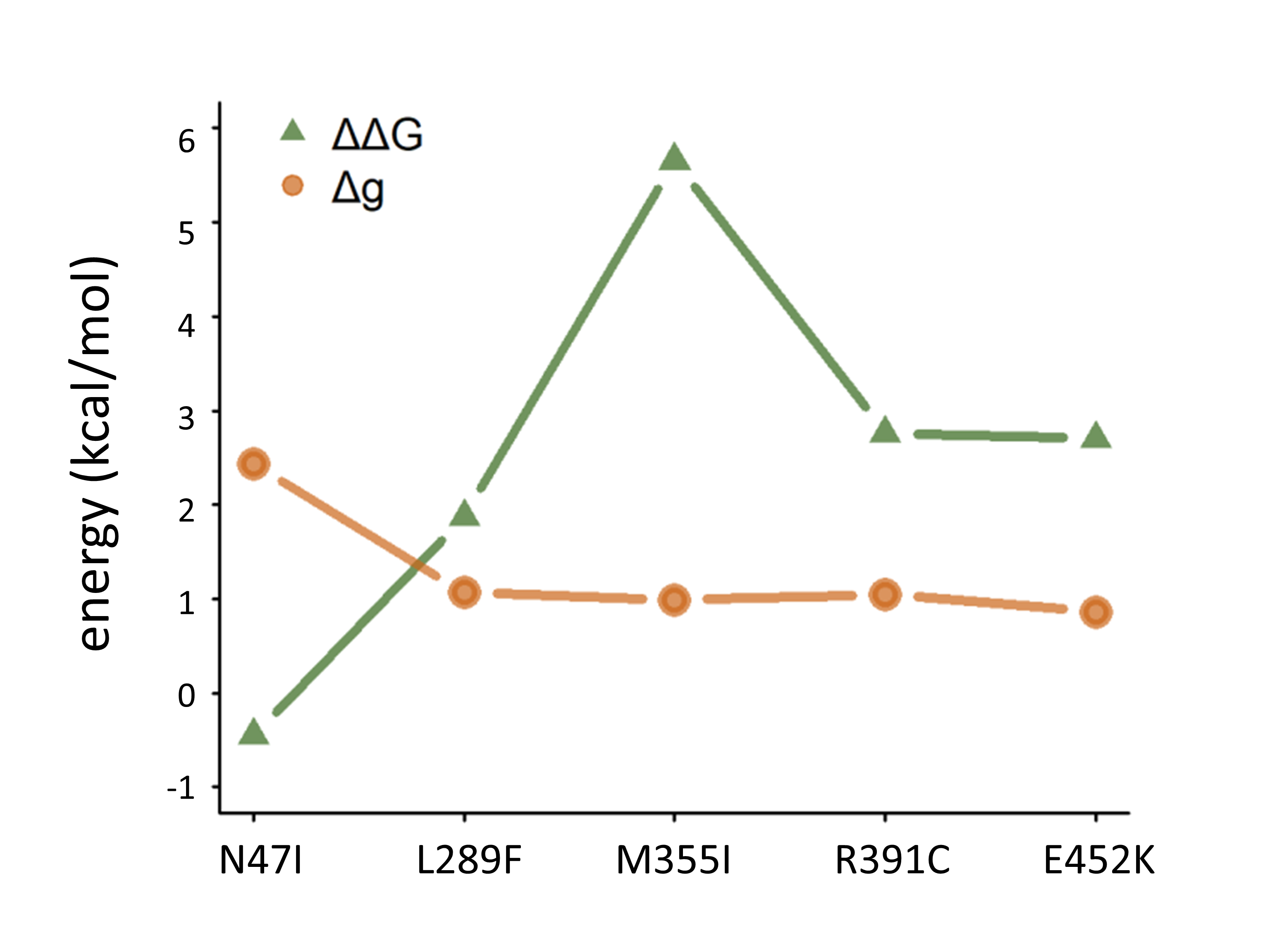

Supplement: S8 Fig — Graphical comparison of ΔΔG (the green line) and Δg (the orange line) for the choose severe mutations. (TIF) [file pcbi.1010009.s008.tif]
