# Supplementary material for: Dissecting mutational allosteric effects in alkaline phosphatases associated with different Hypophosphatasia phenotypes: An integrative computational investigation
Source: PLoS Comput Biol. 2022 Mar 23;18(3):e1010009. doi: 10.1371/journal.pcbi.1010009 (PMC8979438; doi:10.1371/journal.pcbi.1010009)
Supplement: S1 Text — (DOCX) [file pcbi.1010009.s010.docx]

**S1 Text.**

**Sequence and Structural Landscape of ALPL mutations.** Overall, 185 missense mutations related to 142 unique amino acids were found in the *ALPL* gene, of which 111 mutations related to 100 residues were found to cause the mild form of HPP, while 74 mutations related to 63 residues were found to cause the severe form of HPP (Fig 2A). Moreover, 57 mutations related to 55 residue sites were found in asymptomatic people and, therefore, served as the control group. It should be noted that a residue may be related to different types of mutations (S1 Fig), while the Venn Diagram showed overlap of mutated residues of all three groups (Fig 2A). Structurally (Fig 2C), it was found that 104 out of 185 (66%) pathogenic mutations were located at these five functional domains; in particular, 17% were at the crown domain, while only 28 of 57 (49%) mutations in the control group were distributed in this domain. In the mild group, 12 mutations were located at the active center, 21 at the interface, 23 at the crown domain, 9 at the Ca^2+^ binding site, and 3 at the N-terminus. The distribution of mutations in the severe group was as follows: 7 mutations at the active center, 4 at the interface, 9 at the crown domain, 12 at the Ca^2+^ binding site, and 4 in the N-terminus (Fig 2D). On the other hand, in the control group, 16 mutations were located at the Ca^2+^ binding site, 2 at the interface, 6 at the crown domain and 4 at the N-terminus.
