# Supplementary material for: Dissecting mutational allosteric effects in alkaline phosphatases associated with different Hypophosphatasia phenotypes: An integrative computational investigation
Source: PLoS Comput Biol. 2022 Mar 23;18(3):e1010009. doi: 10.1371/journal.pcbi.1010009 (PMC8979438; doi:10.1371/journal.pcbi.1010009)
Supplement: S2 Text — (DOCX) [file pcbi.1010009.s011.docx]

**S2 Text.**

# Machine Learning Models for Feature Selection. The random forest (RF) model was developed by using an RF algorithm via the R package caret and random forest. A typical RF model consists of hundreds of decision trees and uses majority voting to determine the final prediction outcome for unseen data samples; it allows selection of variables or features, thereby providing an opportunity to characterize the important features that contribute the most to the performance of the model.[[1](#_ENREF_1)] We used 70% of the whole dataset as the training set to perform machine learning, and the remaining 30% for model assessment. Both had the same distribution of mild, severe and control groups as the total dataset. Meanwhile, in the process of training, we applied Recursive Feature Elimination (RFE) algorithm using the caret package,[[2](#_ENREF_2)] which starts from a model with all features and, in each iteration, drops out a certain number of least important features, with 10-fold cross-validation, to delete the uninformative predictors in case of over-fitting issues. The feature importance evaluation standard mean decrease accuracy is the degree of decrease in the accuracy of RF prediction by changing the value of a variable to a random number. The larger the value, the greater the importance of the variable. To assess the performance of the RF model, accuracy was calculated to measure the performance of the classification model, which is calculated as:

$\mathrm{Accuracy}=\frac{TP+TN}{\mathrm{All}}$ (1)

The true positives (TP) and true negatives (TN) are defined as the number of mutations that are classified correctly as severe, mild, and neutral mutations, respectively. Likewise, false positives (FP) and false negatives (FN) are defined as the number of mutations that are misclassified into the other mutational classes. The importance evaluation standard of a single feature between two groups is the area under the curve (AUC), which is calculated by the R package pROC.[[3](#_ENREF_3)] It represents the area under the receiver operating characteristic (ROC) curve, which is a plot where sensitivity is plotted as a function of 1-specificity. The sensitivity and specificity are defined as:

$\mathrm{Sensitivity}=\frac{\mathrm{TP}}{TP+FN}$ (2)

$\mathrm{Specificity}=\frac{\mathrm{TN}}{TN+FP}$ (3)

**REFERENCES**

1. Song J, Li F, Takemoto K, Haffari G, Akutsu T, Chou K-C, et al. PREvaIL, an integrative approach for inferring catalytic residues using sequence, structural, and network features in a machine-learning framework. Journal of theoretical biology. 2018;443:125-137. doi: 10.1016/j.jtbi.2018.01.023

2. Guyon I, Weston J, Barnhill S, Vapnik V. Gene selection for cancer classification using support vector machines. MACHINE LEARNING. 2002;46(1-3):389-422. doi: 10.1023/A:1012487302797

3. Robin X, Turck N, Hainard A, Tiberti N, Lisacek F, Sanchez J-C, et al. pROC: an open-source package for R and S plus to analyze and compare ROC curves. BMC BIOINFORMATICS. 2011;12. doi: 10.1186/1471-2105-12-77
